# *BMP7* expression in mammalian cortical radial glial cells increases the length of the neurogenic period

**DOI:** 10.1093/procel/pwad036

**Published:** 2023-06-10

**Authors:** Zhenmeiyu Li, Guoping Liu, Lin Yang, Mengge Sun, Zhuangzhi Zhang, Zhejun Xu, Yanjing Gao, Xin Jiang, Zihao Su, Xiaosu Li, Zhengang Yang

**Affiliations:** State Key Laboratory of Medical Neurobiology and MOE Frontiers Center for Brain Science, Institutes of Brain Science, and Department of Neurology, Zhongshan Hospital, Fudan University, Shanghai 200433, China; State Key Laboratory of Medical Neurobiology and MOE Frontiers Center for Brain Science, Institutes of Brain Science, and Department of Neurology, Zhongshan Hospital, Fudan University, Shanghai 200433, China; State Key Laboratory of Medical Neurobiology and MOE Frontiers Center for Brain Science, Institutes of Brain Science, and Department of Neurology, Zhongshan Hospital, Fudan University, Shanghai 200433, China; State Key Laboratory of Medical Neurobiology and MOE Frontiers Center for Brain Science, Institutes of Brain Science, and Department of Neurology, Zhongshan Hospital, Fudan University, Shanghai 200433, China; State Key Laboratory of Medical Neurobiology and MOE Frontiers Center for Brain Science, Institutes of Brain Science, and Department of Neurology, Zhongshan Hospital, Fudan University, Shanghai 200433, China; State Key Laboratory of Medical Neurobiology and MOE Frontiers Center for Brain Science, Institutes of Brain Science, and Department of Neurology, Zhongshan Hospital, Fudan University, Shanghai 200433, China; State Key Laboratory of Medical Neurobiology and MOE Frontiers Center for Brain Science, Institutes of Brain Science, and Department of Neurology, Zhongshan Hospital, Fudan University, Shanghai 200433, China; State Key Laboratory of Medical Neurobiology and MOE Frontiers Center for Brain Science, Institutes of Brain Science, and Department of Neurology, Zhongshan Hospital, Fudan University, Shanghai 200433, China; State Key Laboratory of Medical Neurobiology and MOE Frontiers Center for Brain Science, Institutes of Brain Science, and Department of Neurology, Zhongshan Hospital, Fudan University, Shanghai 200433, China; State Key Laboratory of Medical Neurobiology and MOE Frontiers Center for Brain Science, Institutes of Brain Science, and Department of Neurology, Zhongshan Hospital, Fudan University, Shanghai 200433, China; State Key Laboratory of Medical Neurobiology and MOE Frontiers Center for Brain Science, Institutes of Brain Science, and Department of Neurology, Zhongshan Hospital, Fudan University, Shanghai 200433, China

**Keywords:** radial glia, cortical neurogenesis, cortical gliogenesis, cortical evolution, BMP7, SHH

## Abstract

The seat of human intelligence is the human cerebral cortex, which is responsible for our exceptional cognitive abilities. Identifying principles that lead to the development of the large-sized human cerebral cortex will shed light on what makes the human brain and species so special. The remarkable increase in the number of human cortical pyramidal neurons and the size of the human cerebral cortex is mainly because human cortical radial glial cells, primary neural stem cells in the cortex, generate cortical pyramidal neurons for more than 130 days, whereas the same process takes only about 7 days in mice. The molecular mechanisms underlying this difference are largely unknown. Here, we found that bone morphogenic protein 7 (BMP7) is expressed by increasing the number of cortical radial glial cells during mammalian evolution (mouse, ferret, monkey, and human). *BMP7* expression in cortical radial glial cells promotes neurogenesis, inhibits gliogenesis, and thereby increases the length of the neurogenic period, whereas Sonic Hedgehog (SHH) signaling promotes cortical gliogenesis. We demonstrate that BMP7 signaling and SHH signaling mutually inhibit each other through regulation of GLI3 repressor formation. We propose that *BMP7* drives the evolutionary expansion of the mammalian cortex by increasing the length of the neurogenic period.

## Introduction

Mammalian cortical radial glial (RG) cells are the source for all cortical pyramidal neurons (PyNs), most cortical glial cells, and a subpopulation of olfactory bulb interneurons (Rakic, 2006; Kriegstein and Alvarez-Buylla, 2009; Molnar et al., 2019; Lin et al., 2021). Three types of RG cells drive human cortical development; they are ventricular zone (VZ) full span radial glia (fRG), VZ truncated radial glia (tRG), and outer radial glia (oRG) which lie in the outer subventricular zone (OSVZ) (LaMonica et al., 2013; Nowakowski et al., 2016; Baburamani et al., 2020; Yang et al., 2022). Human cortical neuroepithelial cells start to convert into fRG cells around gestational week 7 (GW7), and then fRG cells undergo asymmetric cell division to self-renew and to produce PyN intermediate progenitor cells (IPCs) around GW8, which exclusively differentiate into cortical deep layer PyNs. After 9 weeks of neurogenesis, around GW16–GW17, human cortical fRG cells give rise to oRG and tRG cells. oRG cells inherit long basal fibers of fRG cells, while tRG cells inherit apical domains of fRG cells (LaMonica et al., 2013; Nowakowski et al., 2016; Yang et al., 2022). Both oRGs and tRGs can self-renew. The emergence of the cortical OSVZ in higher-order mammals is fundamentally different from mice (Smart et al., 2002). oRG cells in the OSVZ mainly generate upper layer cortical PyNs (Lui et al., 2011; Molnar et al., 2019; Cardenas and Borrell, 2020), and a recent study suggests that tRG cells in the VZ primarily generate cortical glia (Yang et al., 2022). After tRG cells are generated from fRG cells, a subpopulation of putative primed tRG cells expresses ASCL1 and epidermal growth factor receptor (EGFR). Initially, this population of primed tRG cells generates PyNs, but then they undergo a neurogenesis-to-gliogenesis switch, and generate basal multipotent intermediate progenitor cells (bMIPCs) that express ASCL1, EGFR, and oligodendrocyte transcription factor 2 (OLIG2) (Yang et al., 2022). These bMIPCs, at the population level, undergo several rounds of mitosis and give rise to most of the cortical glial cells (oligodendrocytes and astrocytes) and a subpopulation of olfactory bulb interneurons (Yang et al., 2022). It is worth noting that this process in the human cortex is similar to that in the mouse cortex (Li et al., 2021), suggesting that mammalian cortical gliogenesis is evolutionarily conserved, and that the presence of EGFR-expressing progenitors in the cortical VZ/SVZ is a strong signal for the onset of cortical gliogenesis (Fu et al., 2021; Li et al., 2021; Yang et al., 2022).

On the other hand, humans have a longer neurogenic period compared to perhaps all other mammals (Lewitus et al., 2014). This allows human cortical RG cells to undergo more cell division and therefore produce more neurons (Lewitus et al., 2014; Picco et al., 2018; Stepien et al., 2021). However, how the length of the neurogenic period is regulated in the mammalian brain remains a fundamental unanswered question.

## Results

### Human cortical oRG cells are neurogenic while tRG cells are gliogenic

**Figure 1.**
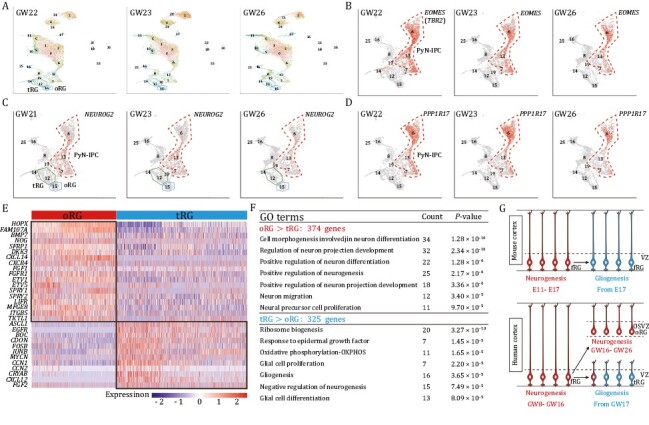
**Human cortical oRG cells and tRG cells exhibit distinct transcriptional signatures.** (A–D) scRNA-Seq analysis of molecular profiles of human cortical cells at GW22, GW23, and GW26. tSNE plot showed *EOMES (TBR2)*-, *NEUROG2*-, and *PPP1R17*-expressing PyN-IPCs, suggesting that cortical neurogenesis extends to GW26. (E) Heat map of selected differentially expressed genes for oRG cells versus tRG cells. Note that *HOPX* and *FAM107A* are enriched in oRG cells. (F) Selected GO terms implicating oRG cells are neurogenic while tRG cells are gliogenic. (G) Schematic summarizing mouse and human cortical RG cell lineage progression. Note that the human OSVZ is a duplicated neurogenic zone, with a greatly prolonged period of cortical neurogenesis. Thus, the length of the cortical neurogenic period is more than 130 days (GW8–GW26) in humans.

Recent studies reported that human cortical neurogenesis ceases around GW19 (Pebworth et al., 2021), and reported that cortical oRG cells start to generate IPCs of oligodendrocytes, astrocytes, and interneurons around GW20 (Huang et al., 2020; Allen et al., 2022; Liu et al., 2023). In contrast, we found that human cortical neurogenesis extends to GW26, based on analyzing published single-cell RNA sequencing (scRNA-Seq) data of human GW22 (12,567 cells), GW23 (12,557 cells), and GW26 (13,371 cells) cortical tissue (Figs. 1A–D, S1A; Table S1) (Trevino et al., 2021). PyNs are continuously produced by PyN-IPCs that expressed *EOMES* (*TBR2*), *NEUROG2*, *PPP1R17*, *PAX6*, *NEUROG1*, *NEUROD4*, *INSM1*, *SSTR2*, *ELAVL2*, *ELAVL4*, and *PENK* (Figs. 1A–D and S1A–C) (Pollen et al., 2015; Girskis et al., 2021). This result is consistence with our previous immunohistochemical analyses on GW23 human cortical samples (Yang et al., 2022).

To define the major transcriptional features that underlie neurogenesis versus gliogenesis in the developing human cortex, we performed differential gene expression analysis between oRG cells and tRG cells in GW22–GW26 cortex. Although both human cortical oRG and tRG cells are derived from fRG cells, they exhibit distinct transcriptional signatures; oRG cells had 374 genes while tRG cells had 325 genes that were expressed at relatively higher levels (Table S1). Selected differentially expressed genes are shown in the heat map (Fig. 1E). Gene Ontology (GO) analysis of differentially expressed genes revealed that, in general, cortical oRG cells are neurogenic while tRG cells are highly gliogenic (Fig. 1F). Briefly, compared to tRG cells, oRG cells expressed higher levels of *BMP7*, *HOPX*, *LIFR*, *CXCL14*, *MFGE8*, *NOG* (*NOGGIN*), *TKTL1*, integrin *ITGB5,* and WNT pathway inhibitors (*SFRP1* and *DKK3*) (Fig. 1E). In addition, ~30% of oRG cells expressed *FGF2* (Fig. S1D). Furthermore, FGF/MAPK signaling is elevated in oRG cells based on their expression of *FGFR1*, *ETV1*, *ETV5*, *SPRY1,* and *SPRY2* (Fig. 1E). We speculate that FGF2/MAPK activity in oRGs is a cell-autonomous mechanism that antagonizes autocrine quiescent bone morphogenic protein 7 (BMP7) signaling, through phosphorylation of the SMAD linker domain (Kretzschmar et al., 1997; Pera et al., 2003). Indeed, previous studies showed that while BMP signaling alone promotes terminal astrocytic differentiation, exposure to both BMP and FGF2 maintains the stem cell character of cultured progenitor cells (Sun et al., 2011; Martynoga et al., 2013).

### Signaling pathways driving gliogenesis in tRG cells

PyN-IPCs derived from cortical neurogenic RG cells are slow-cycling cells (Girskis et al., 2021). In contrast, bMIPCs derived from cortical gliogenic tRG cells are rapid cycling (Li et al., 2021; Yang et al., 2022). We identified six molecular programs in tRG cells that drive gliogenesis: (i) tRG cells are in the cortical VZ, so their primary cilia contact cerebrospinal fluid (CSF). Sonic Hedgehog (SHH), a morphogen and mitogen, is in the CSF (Huang et al., 2009; Lun et al., 2015; Winkler et al., 2018). Furthermore, tRG cells express higher levels of *BOC* and *CDON* than oRG cells (Fig. 1E); they are coreceptors of *PTCH1* that enhance SHH signaling (Allen et al., 2011). (ii) Transcription effects of SHH signaling are mediated by GLI transcription factors. In the absence of SHH signaling, GLI3 full-length (GLI3FL) protein is processed to a truncated repressor form of GLI3 (GLI3R) that represses SHH target genes. This process depends on adenylyl cyclase-mediated cAMP-dependent protein kinase A (PKA), which phosphorylates GLI3FL, stimulating the proteolytic processing of GLI3FL to GLI3R. Upon SHH pathway activation, the SHH signaling cascade decreases PKA activity, prevents GLI3R production, and promotes the formation of GLI activators that drive transcription of SHH target genes (Wang et al., 2000; Hui and Angers, 2011). We found that 50% of human cortical tRG cells expressed *CXCL12* (Fig. 1E), whereas very few mouse cortical RG cells expressed *Cxcl12*. *CXCR4*, expressed by a subset of human cortical RG cells (Figs. 1E and S1E), is a G protein–coupled receptors (GPCR) that activates Gαi protein and inhibits cAMP-PKA signaling. Thus, the autocrine activation of CXCR4 in human tRG cells further decreases GLI3R production, thereby increasing SHH signaling (Klein et al., 2001). CXCL12/CXCR4 also activates PI3K/AKT/mTOR and YAP-CCN1/2 signaling (Rao et al., 2016; Walenkamp et al., 2017; Shi et al., 2020). (iii) About 30% of tRG cells express FGF2, and most of them express FGFR1 and FGFR2 (Figs. 1E and S1D), thus FGF/MAPK pathway is also activated in tRG cells. (iv) Ribosomal biosynthesis genes are critical targets of cellular pro-growth signaling (Pelletier et al., 2018), and we found that more than 70 large and small ribosomal subunit genes were significantly upregulated in tRG cells (Fig. S2). (v) tRG cells also express higher levels of several key oxidative phosphorylation (OXPHOS) components (*MT-ND1-5* and *MT-CO1-3*) (Fig. S2), suggesting their preferential use of OXPHOS instead of glycolysis (Fig. 1F). These data also indicate a metabolic shift from glycolysis to OXPHOS when cortical RG cells become gliogenic. (vi) Most importantly, ~40% of tRG cells are primed/poised for generating bMIPCs, as they express ASCL1 and EGFR (Fig. 1E), which then give rise to bMIPCs that express ASCL1, EGFR, and OLIG2 (Fig. S1F) (Yang et al., 2022). SHH signaling through *Smo* is required in cortical RG cells for gliogenesis (Winkler et al., 2018; Zhang et al., 2020b), as is MAPK signaling (Li et al., 2012, 2014). Both are also required for cortical RG cell proliferation (Komada et al., 2008; Lavoie et al., 2020). These data suggest that when proliferative and gliogenic signals (SHH + FGF/MAPK) overcome the threshold of quiescent and neurogenic signals (GLI3R + BMP7), tRG cells switch from neurogenesis to gliogenesis; this process in humans takes place over a much longer period than that in the mouse cortex (Yang et al., 2022).

Taken together, our analysis reveals that human cortical neurogenesis extends to at least GW26, due to the maintenance of the oRG cells’ capacity to generate PyNs, and not due to tRG cells. The human neocortex has two germinal zones from GW16 to GW26: the gliogenic VZ and the neurogenic OSVZ (Fig. 1G) (Yang et al., 2022). This ensures that the onset of cortical gliogenesis normally occurs in the VZ around GW17, while oRGs continue to produce neurons in the OSVZ, thus increasing the length of the neurogenic period for another 10 weeks (GW16–GW26) (Fig. 1G).

### An evolutionary increase in *BMP7* expression in mammalian cortical RG cells

**Figure 2.**
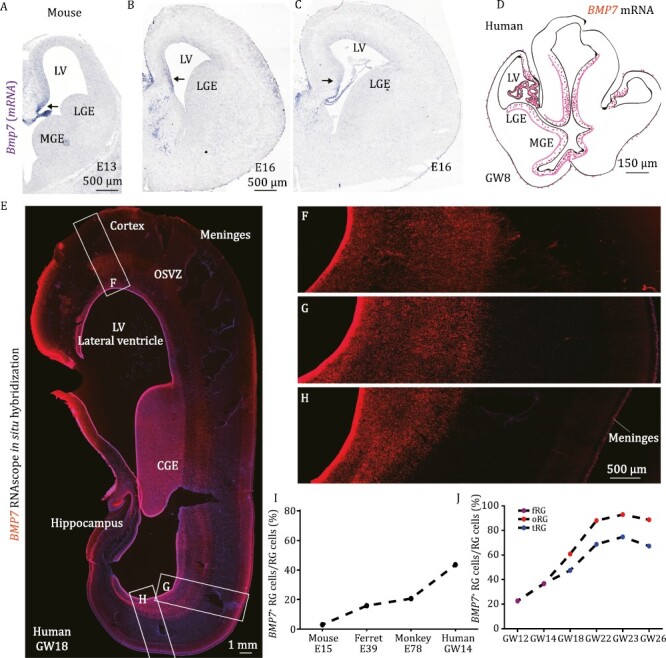
***BMP7* is expressed by more and more cortical RG cells during mammalian development and evolution.** (A–C) *In situ* hybridization shows that *Bmp7* mRNA is expressed in the mouse medial cortex, but not in the dorsal cortex at E13.0 and E16.0. (D) Maps of *BMP7-*expressing cells in the human cortex at GW8. The original picture of *BMP7* mRNA *in situ* hybridization on human GW8 cortical section is from previous studies (Abu-Khalil et al., 2004). (E–H) RNAscope *in situ* hybridization shows *BMP7* expression in germinal zones of the human cortex and caudal ganglionic eminence (CGE) at GW18. Note *BMP7* is also expressed in meninges. (I) Comparative scRNA-Seq analyses reveal an increase in the percentage of cortical RG cells that expressed *BMP7* in the E15 mouse cortex, E39 ferret dorsal cortex, E78 rhesus monkey visual cortex, and GW14 human prefrontal cortex. (J) Increasing the percentage of human cortical RG cells that expressed *BMP7* with increasing gestational age. We define a *BMP7*-expressing cell that expressed at least one UMI count. To compare UMI counts across samples, we took into account differences in sequencing depth between the samples according to the normalization procedure provided by 10× genomic. LGE, lateral ganglionic eminence; MGE, medial ganglionic eminence.

*BMP7* is enriched in human but not in mouse cortical RG cells (Lui et al., 2014), and it is expressed relatively higher in oRG cells than that in tRG cells (Figs. 1E and S1D). We examined *BMP7* expression in different mammalian cortical RG cells by integrating the analysis of scRNA-Seq datasets with mRNA *in situ* hybridization experiments. We found that between stages of cortical patterning and the onset of cortical neurogenesis, *BMP7* expression pattern shows a startling similarity between mouse and human: *BMP7* is expressed by the choroid plexus and cortical hem, and weak *BMP7* expression extends to the medial cortex (Fig. 2A–D) (Abu-Khalil et al., 2004). Soon after, the growth of the human telencephalon accelerates, *BMP7* expression begins in human cortical RG cells, whereas very few mouse dorsal cortical RG cells express *Bmp7* (Fig. 2E–H). Comparative scRNA-Seq analyses reveal that there is an increase in *BMP7* expression by cortical neurogenic fRG cells of mouse at E15, ferret at E39, rhesus monkey at E78, and human at GW14 (Figs. 2I, S3 and Table S2). As human development proceeds from GW12 to GW26, more cortical RG cells express *BMP7* (from ~20% to ~90%) (Figs. 2J, S4 and Table S3), as well as BMP receptors *BMPR1A*, *BMPR1B,* and *BMPR2* (Fig. S5 and Table S3) (Panchision et al., 2001; Hebert et al., 2002; Caronia et al., 2010). These results suggest the importance of *BMP7* during mammalian cortical development and evolution.

### BMP7 and SHH signaling inhibit each other to regulate mouse cortical gliogenesis

**Figure 3.**
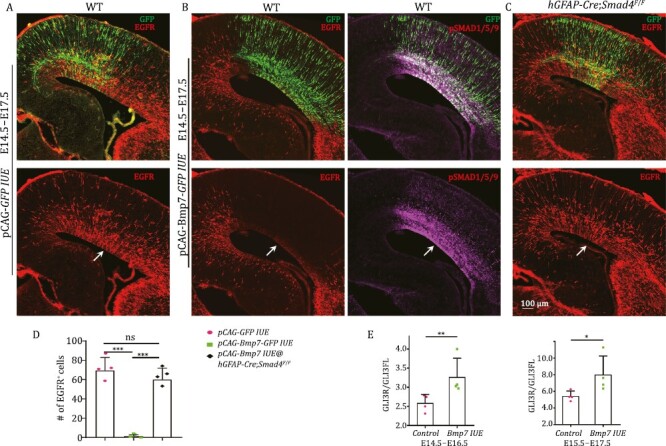
**BMP7-SMAD signaling inhibits EGFR expression.** (A and B) IUE of vectors that express either *GFP* (control) or *Bmp7-GFP* in E14.5 WT mouse cortex. At E17.5, EGFR expression was significantly downregulated and pSMAD1/5/9 expression was strongly upregulated in the *Bmp7*-IUE cortex (arrows). (C) In the *hGFAP-Cre*;*Smad4*^*F*/*F*^ cortex, *Bmp7*-IUE showed less EGFR inhibition. (D) Quantification of numbers of EGFR-expressing cells in the cortex. (E) *Bmp7* was overexpressed in the E14.5 or E15.5 cortex using IUE. The cortical GLI3R to GLI3FL ratio was significantly increased 48 h later (see Western blot in Fig. S8).

Because very few mouse dorsal cortical RG cells express *Bmp7*, we first explored its function using a gain-of-function method. *Bmp7* was delivered (via *pCAG-Bmp7-Gfp*) into the mouse dorsal cortical VZ at E14.5 using *in utero* electroporation (IUE). Controls were electroporated with *pCAG-GFP*. At E17.5, while the control cortex expressed strong EGFR (Fig. 3A), the *pCAG-Bmp7-Gfp*-IUE cortex did not (Fig. 3B). BMP receptor signaling triggers the phosphorylation and activation of SMAD1/5/9 proteins, which form a complex with SMAD4 that together regulates gene expression (Wang et al., 2014b). Accordingly, cortical *BMP7* overexpression resulted in strong expression of pSMAD1/5/9 and their downstream targets ID3, ID1, and HOPX (Figs. 3B and S6). However, in the absence of cortical *Smad4* function (*hGFAP-Cre*;*Smad4*^F/F^ mice), *Bmp7* overexpression did not inhibit EGFR expression (Fig. 3C and 3D). EGFR is autonomously required for cortical gliogenesis (Zhang et al., 2020a, 2023). Thus, BMP7–SMAD signaling inhibits cortical gliogenesis probably by downregulating EGFR expression.

We next examined BMP function in the medial cortex by overexpressing a repressor of BMP signaling. *hGFAP-Cre* is active in medial cortical RG cells from E12 (Fig. S7A and S7B); it was used to overexpress BMP inhibitor NOG (using *Nog*^*OE*/+^ mice). At E16.5, while EGFR expression was weak in the medial cortex of *hGFAP-Cre* control mice, its expression was upregulated in the medial and/or dorsal cortex of *hGFAP-Cre*;*Nog*^*OE*/+^ mice (Fig. S7C). In contrast, ID3 and pSMAD1/5/9 expression were downregulated in the medial cortex (Fig. S7C). Thus, BMP signaling inhibits gliogenesis mainly in the mouse medial cortex. Interestingly, the mouse medial cortex has human oRG-like cells (Vaid et al., 2018); perhaps, this is due to *Bmp7* expression.

BMPs and SHH are known to antagonize the downstream response to each other (Ulloa and Briscoe, 2007; Hebert and Fishell, 2008; Hoch et al., 2009). However, mechanisms for this remain elusive. We examined whether BMP7 signaling upregulates cortical GLI3R expression. *Bmp7* was electroporated into the mouse cortex at E14.5 or E15.5; the *Bmp7*-IUE cortex was collected 48 h later for GLI3 Western blot assay. *Bmp7* overexpression significantly increased the GLI3R/GLI3FL ratio in the cortex mainly due to excess production of GLI3R (Fig. 3E; see Western blot in Fig. S8), which antagonizes SHH signaling.

**Figure 4.**
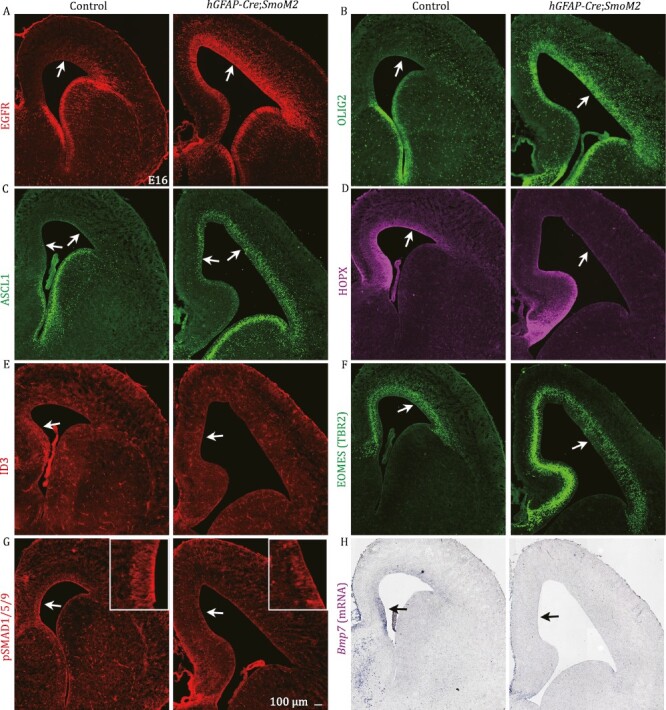
**Constitutively active *SmoM2* inhibits BMP7 signaling and promotes cortical gliogenesis.** (A–H) The expression of EGFR, OLIG2, and ASCL1 was increased, whereas the expression of HOPX, ID3, EOMES (TBR2), pSMAD1/5/9, and *Bmp7* was decreased in the medial and/or dorsal cortex of *hGFAP-Cre*;*SmoM2* mice compared to control mice at E16 (arrows).

Next, we ectopically expressed non-cholesterol-modified SHH (SHHN) in the cortical VZ by IUE of *pCAG-ShhN-GFP* into wild-type (WT) mice at E15.5 and examined the cortex at E16.5. The increased *ShhN* expression upregulated EGFR, ASCL1, and OLIG2 expression compared to the *pCAG-GFP*-IUE (control) cortex (Fig. S9A–F). In contrast, ID3 and HOPX expression were downregulated upon *ShhN* overexpression (Fig. S9G–K). Expression of a constitutively active SMO protein that drives SHH signaling (*hGFAP-Cre*;*SmoM2* mice) also increased in EGFR, ASCL1, and OLIG2 expression (Fig. 4A–C), and decreased in *Bmp7*, pSMAD1/5/9, ID3, HOPX, and EOMES expression in the E16.0 medial and/or dorsal cortex (Fig. 4D–H). Together, these results indicate that increased SHH signaling inhibits *Bmp7* expression in the cortex and promotes cortical gliogenesis.

**Figure 5.**
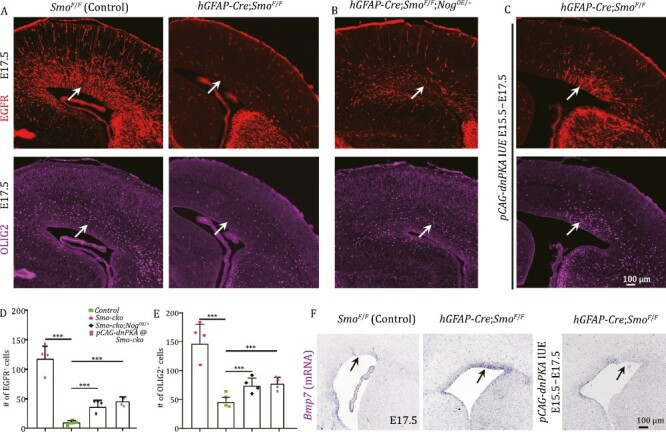
**SHH-SMO signaling is required for cortical gliogenesis.** (A) The expression of EGFR and OLIG2 was severely downregulated in the E17.5 cortex of *hGFAP-Cre*;*Smo*^*F*/*F*^ mice (*Smo-cko*). (B and C) Overexpression of *Nog* or *dnPKA* largely rescued the downregulation of EGFR and OLIG2 (arrows). (D and E) Quantification of numbers of cortical EGFR- and OLIG2-expressing cells. (F) *Bmp7* mRNA expression was upregulated in the E17.5 cortex of *hGFAP-Cre*;*Smo*^*F*/*F*^ mice, but *dnPKA*-IUE blocked *Bmp7* upregulation.

Then we tested whether EGFR expression in the dorsal cortex requires SHH signaling by deleting *Smo* (*hGFAP-Cre*;*Smo*^*F*/*F*^ mice). At E17.5, very few EGFR- and OLIG2-expressing cortical cells were detected in *hGFAP-Cre*;*Smo*^*F*/*F*^ mice (Fig. 5A); their expression partially recovered by P2 (Fig. S10). This result suggests that, without SHH signaling, cortical gliogenesis is greatly retarded.

To understand the molecular mechanism responsible for SHH-mediated gliogenesis, we examined *Bmp7* expression. mRNA *in situ* hybridization revealed that *Bmp7* expression was increased in the cortical VZ of *hGFAP-Cre*;*Smo*^*F*/*F*^ mice at E17.5 (Fig. 5F). Consistent with this observation, expression of BMP7 effector pSMAD1/5/9, ID3 and HOPX was upregulated (Fig. S11). We also examined the E17.5 cortex of *hGFAP-Cre*;*Smo*^*F*/*F*^; *Nog*^*OE*/+^ mice and found that EGFR and OLIG2 expression were partially rescued (Fig. 5B), supporting the idea that upregulation BMP7 signaling contributes to downregulation of EGFR and OLIG2 expression in the cortex of *hGFAP-Cre*;*Smo*^*F*/*F*^ mice.

Previous studies have demonstrated that the dorsal cortical identity and neurogenesis are protected by GLI3R-mediated maintaining *Bmp* and *Wnt* expression, and suppression from the ventralizing effects of SHH and FGF signaling (Tole et al., 2000; Aoto et al., 2002; Kuschel et al., 2003; Rash and Grove, 2007). To investigate the function of GLI3R, we used *hGFAP-Cre*;*Gli3*^*F*/*F*^ mice. We examined E16.0 cortex and found that EGFR and OLIG2 expression were increased (Fig. S12). This result suggests that, in addition to BMP7, GLI3R also represses EGFR and OLIG2 expression. Furthermore, the GLI3R/GLI3FL ratio was significantly increased in the cortex of *hGFAP-Cre*;*Smo*^*F*/*F*^ mice (Fig. S8A). We therefore blocked the generation of GLI3R by ectopically expressing a dominant negative form of PKA (*dnPKA*). In the WT cortex that was electroporated with *dnPKA*, upregulation of EGFR, OLIG2, and ASCL1 was observed (Fig. S13). Similarly, in the cortex of *hGFAP-Cre*;*Smo*^*F*/*F*^ mice, we observed a rescue of EGFR, OLIG2, and ASCL1 expression in the *dnPKA* electroporated side compared to the contralateral side (Figs. 5C–E and S14). Moreover, *Bmp7* upregulation disappeared upon dnPKA expression (Fig. 5F). This result suggests that an increase in GLI3R production could be key to inducing *Bmp7* expression in the dorsal cortex of *hGFAP-Cre*;*Smo*^*F*/*F*^ mice. In other words, SHH signaling promotes cortical progenitor proliferation and gliogenesis by reducing GLI3R production, which also contributes to preventing *Bmp7* expression to spread from the medial cortex to the dorsal cortex.

### Increase in BMP*7* signaling and loss of SHH signaling prolong mouse cortical neurogenic period

**Figure 6.**
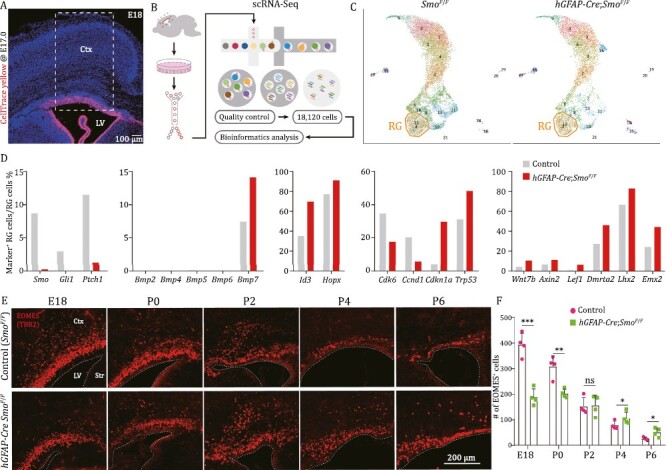
**Loss of SHH-SMO signaling in mouse cortical RG cells lengthens their neurogenic period.** (A–C) Schematic of the workflow of scRNA-Seq analysis. CellTrace Yellow was injected into the lateral ventricle of *Smo*^*F*/*F*^ or *hGFAP-Cre*;*Smo*^*F*/*F*^ mice at E17.0. The cortex was collected at E18.0 for cell sorting and scRNA-Seq analysis. (D) Loss of *Smo* function in cortical RG cells resulted in significantly downregulation of *Smo*, *Gli1*, *Ptch1*, and upregulation of *Bmp7*, *Id3,* and *Hopx*, and inhibition of cell proliferation (downregulation of *Cdk6* and *Ccnd1*, and upregulation of *Cdkn1a* and *Trp53*). A subset of WNT and BMP response neurogenic genes (*Wnt7b*, *Axin2*, *Lef1*, *Dmrta2*, *Lhx2*, and *Emx2*) was upregulated in cortical RG cells. (E and F) Numbers of EOMES-expressing cells were decreased in the E18 and P0 cortex, whereas increased in the P4 and P6 cortex of *hGFAP-Cre*;*Smo*^*F*/*F*^ mice, compared to *Smo*^*F*/*F*^ (control) mice, indicating that the length of the cortical neurogenic period is increased. Ctx, cortex; LV, lateral ventricle; Str, striatum.

Finally, we performed scRNA-Seq transcriptome analysis of cortical cells in *hGFAP-Cre*;*Smo*^*F*/*F*^ mice and littermate *Smo*^*F*/*F*^ control mice at E18.0 (Fig. 6A–C and Table S4). First, we identified that expression of *Smo*, *Gli1,* and *Ptch1* was in the ground state in the cortex of *hGFAP-Cre*;*Smo*^*F*/*F*^ mice (Figs. 6D and S15A). Second, we confirmed that *Bmp7*, but not *Bmp2, Bmp4, Bmp5,* or *Bmp6* (Fig. 6D), was upregulated in cortical RG cells without SHH-SMO function. BMP7 response genes *Id3* and *Hopx* were also upregulated (Fig. 6D). Third, cell proliferation was greatly reduced because the expression of *Cdk6* and *Ccnd1* was significantly downregulated while *Cdkn1a* (P21) and *Trp53* were upregulated (Fig. 6D). Fourth, the generation of *Egfr*-, *Olig1*-, and *Olig2*-expressing bMIPCs was greatly reduced (Fig. S15B), consistent with immunostaining results (Fig. 5A). Fifth, a set of WNT and BMP response genes in RG cells that promote cortical neurogenesis was upregulated, including *Wnt7b, Axin2*, *Lef1*, *Dmrta2*, *Lhx2*, and *Emx2* (Figs. 6D and S15A) (Monuki et al., 2001; Theil et al., 2002; Saulnier et al., 2013; Caronia-Brown et al., 2014), strongly indicating that neurogenesis persists in the cortex of *hGFAP-Cre*;*Smo*^*F*/*F*^ mice at E18.0, a time point that cortical RG cells do not undergo neurogenesis in the normal mouse brain. Sixth, consistently, while EOMES-expressing cells (they most likely are PyN-IPCs) in the cortical SVZ were reduced before birth due to reduced cell proliferation (Komada et al., 2008), EOMES-expressing cells increased in the *Smo*-mutant P4 and P6 cortex (Figs. 6E, 6F and S15C). Taken together, these results suggest that, without SHH signaling, cortical RG cell proliferation is greatly reduced and gliogenesis is retarded, but the length of the cortical neurogenic period is increased.

## Discussion

**Figure 7.**
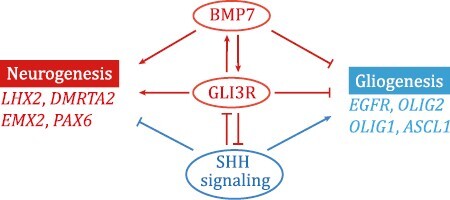
**BMP7 and GLI3R coordinately protect cortical neurogenesis in human brain.** Diagram showing that BMP7/GLI3R and SHH signaling mutually inhibit each other. BMP7 and GLI3R coordinately protect the genetic program of cortical neurogenesis and inhibit cortical gliogenesis. In contrast, SHH signaling promotes cortical gliogenesis and opposes BMP7/GLI3R functions during cortical development.

BMP signaling primarily functions to maintain stem cells in a quiescent state (Li and Clevers, 2010). Here we suggest that BMP7, in combination with cell proliferation signals, sustains the self-renewal of cortical RG cells, maintaining cortical neurogenesis, and inhibiting gliogenesis. On the basis of these findings, we wish to suggest a principle of mammalian cortical development, expansion, and evolution (Fig. 7).

(1) The most recent ancestor to all mammals, i.e., 100 million years ago, is assumed to have a subset of *BMP7*-expressing fRG cells in the dorsal cortex, and is assumed to have already been in some degree gyrencephalic (Fig. S16) (Lewitus et al., 2014). Expression of BMP7 in cortical fRG cells increases GLI3R production, which further strengthens *BMP7* expression in fRG cells. Therefore, *BMP7* expression in cortical fRG cells participates in a positive feedback loop, which antagonizes SHH signaling, protects neurogenesis, inhibits gliogenesis, and thus increases the length of the neurogenic period (Fig. 7). Perhaps, SHH CSF concentration may be reduced in the larger lateral ventricle in bigger-brained mammals; that could further decrease SHH signaling within the dorsal cortex.
(2) Around midterm gestation, the increase in SHH downstream signaling (including CXCL12/CXCR4 signaling), combined with FGF2/MAPK signaling in cortical fRG cells, is probably the major force that drives *BMP7*-expressing cortical fRG cells to give rise to neurogenic oRG and gliogenic tRG cells.
(3) *BMP7* expression in oRG cells also participates in a positive feedback loop. oRG cells in the OSVZ do not contact the lateral ventricle (largely insulated from SHH, similar to cortical RG cells in *hGFAP-Cre*;*Smo*^*F*/*F*^ mice), and express higher level of BMP7 and GLI3R, and maybe never express EGFR (Yang et al., 2022). The more fRG cells express BMP7, the more oRG cells are generated, lengthening the neurogenic period, the more PyNs are generated from the OSVZ, and expanding cortical size during development and perhaps evolution.
(4) Although *BMP7* is highly expressed in oRG cells, *FGF2* and *NOG* expression in oRG cells suppress quiescency and differentiation of BMP7 signaling, thus sustaining self-renew in oRG cells. When *BMP7* expression gets stronger, oRG cells may stop generating PyN-IPCs via asymmetric division, directly transform into astrocytes that also express *BMP7* (Sun et al., 2011), and do not produce oligodendrocyte IPCs (Yang et al., 2022).
(5) On the other hand, for unknown reasons, during development and evolution, there was a subpopulation of mammals (i.e., ancestors of hamsters, mice, and rats) (Fig. S16), whose cortical fRG cells perhaps received higher SHH signaling, which represses *Bmp7* expression in dorsal RG cells, and restricts *Bmp7* expression to the medial cortex. Smaller brains with the smaller lateral ventricle may further increase SHH signaling within the dorsal cortex.
(6) Mouse cortical neurogenesis ceases and gliogenesis occurs when SHH signaling is further increased at ~E17.0. The increase in SHH and MAPK signaling in the mouse dorsal cortical VZ induces a subset of fRG cells to directly transform into cortical astrocyte-IPCs (transforming RG cells) that express EGFR, ASCL1, and OLIG2 (Li et al., 2021), but not oRG cells, as lack of *Bmp7* expression, and induces most cortical RG cells to generate bMIPCs that also express EGFR, ASCL1, and OLIG2 (Li et al., 2021). This population of bMIPCs gives rise to most of the cortical glial cells (oligodendrocytes and astrocytes) and a subpopulation of olfactory bulb interneurons (Yang et al., 2022). Therefore, mouse cortical neurogenesis is mainly protected by Gli3R, whereas human cortical neurogenesis is protected by GLI3R and BMP7; both of them directly or indirectly repress SHH target genes and EGFR expression (Fig. 7).
(7) It is worth noting that *BMP7* expression also extends from the cortex to subcortical ganglionic eminences in higher-order mammalian brains (Fig. 2D–H), thus it may prolong the neurogenic period in most regions of the telencephalon. The study reports that heterozygous mutations in the *BMP7* gene in patients cause small head (Wyatt et al., 2010), providing additional genetic evidence for *BMP7* function in brain development.
(8) BMPs, WNTs, FGFs, and SHH, are used over and over again across ontogeny and phylogeny. In the nervous system, these molecules regulate neural induction, pattern dorsal, ventral, rostral and caudal structures, and regulate neurogenesis versus gliogenesis (Hebert and Fishell, 2008; Hoch et al., 2009). Therefore, it is not surprising that they also play a leading role in driving cortical evolution.
(9) We believe that this basic principle (Fig. 7) is still driving human brain to evolve and to generate a cortex with more neurons.

## Materials and methods

### Animals

All procedures and animal care, including ferrets and mice, were approved and performed in accordance with the Fudan University, Shanghai Medical College Laboratory Animal Research Center guidelines. WT CD-1, *hGFAP-Cre* (Zhuo et al., 2001) (JAX no. 004600), *Smo* flox (Long et al., 2001) (JAX no. 004526), *R26SmoM2* (JAX no. 005130), *Gli3* flox (Blaess et al., 2008) (JAX no. 008873), *Smad4* flox (Yang et al., 2002) (Jax no. 017462), and *pMes-Nog* (Nog overexpression, *Nog*^*OE*/+^) transgenic (Xiong et al., 2009) mice were described previously. The *Nog*^*OE*/+^ mice were generously provided by Professor Yanding Zhang from Fujian Normal University. The day of the vaginal plug detection was designated as Embryonic day 0.5 (E0.5). The day of birth was designated as P0. The genders of the embryonic mice were not determined, and both male and female postnatal mice were used. One pregnant ferret (*Mustela putorius furo*) was purchased from Wuxi Sangosho Biotechnology Co., Ltd, Wuxi, China, and we used one embryo at E39 in this study. The bilateral cortex from one E39 embryo was used for scRNA-Seq.

### Plasmid construction

*pCAG-ires-GFP* plasmid was from Addgene (Addgene #11150), and *pCAG-ShhN-ires-GFP* was used in the previous study (Zhang et al., 2020b). Mouse *Bmp7* cDNA and *dnPKA* cDNA (Clegg et al., 1987) were cloned and inserted into *pCAG-GFP* vector to construct *pCAG-Bmp7-ires-GFP* and *pCAG-dnPKA-ires-GFP* overexpression plasmids.

### *In utero* electroporation (IUE)

Overexpression of plasmids was performed in the cortex using IUE at E14.5 or E15.5. About 0.5 μL of 1–2 μg/μL plasmid solution with 0.05% Fast Green (Sigma) was injected into the lateral ventricle of the embryos using a cable-drawn glass micropipette. Five electrical pulses (duration: 50 ms) were applied across the uterus to E14.5 embryos at 33 V and E15.5 embryos at 35 V. The interval between pulses is 950 ms. Electroporation was performed using a pair of 7 mm platinum electrodes (BTX, Tweezerrode 45-0488, Harvard Apparatus) connected to an electrocleaner (BTX, ECM830).

### Fixation and sectioning of the brain tissue

Embryos were harvested from deeply anesthetized pregnant mice. Each embryo was separated from the placenta, the brain was dissected out, and then fixed in 4% diethylpyrocarbonate and paraformaldehyde (DEPC-PFA) overnight. Postnatal mice were deeply anesthetized and perfused intracardially with 4% paraformaldehyde (PFA). All brains were fixed overnight in 4% PFA at 4°C and cryoprotected in 30% sucrose for at least 24 h, embedded with O.C.T. (Sakura Finetek) in ethanol slush with dry ice. All mouse brains in this study were sectioned in a coronal plane at 20 μm. The GW18 human cortical sections were 60 μm as we used in our previous studies (Ma et al., 2013; Wang et al., 2014a; Yang et al., 2022).

### Immunohistochemistry

All immunohistochemical stains in this study were performed on 20 μm coronal cryostat sections of mouse brains as previously described (Li et al., 2021; Yang et al., 2022). Sections were rinsed with TBS (0.01 mol/L Tris–HCl + 0.9% NaCl, pH = 7.4) for 10 min, incubated in 0.5% Triton-X-100 in TBS for 30 min at room temperature (RT), and then incubated with block solution (5% donkey serum + 0.5% Triton-X-100, pH = 7.2) in TBS for 30 min at RT. For double immunostaining, primary antibodies from different species were incubated simultaneously followed by secondary antibodies. Primary antibodies were diluted in donkey serum block solution and incubated overnight at 4°C, incubated for an additional 30 min at RT, and rinsed three times with TBS. Sections were then incubated with secondary antibodies (1:600, all from Jackson ImmunoResearch) for 2 h at RT, rinsed three times with TBS for 10 min, incubated with 4ʹ,6-diamidino-2-phenylindole (DAPI, 1:5,000, Sigma) diluted in TBS for 5 min, and then finally rinsed three more times with TBS. Primary antibodies used in this study include: goat anti-EGFR (1:1,000, R&D System, BAF1280), rabbit anti-OLIG2 (1:500, Millipore, AB9610), rabbit anti-pSMAD1/5/9 (1:100, Cell signaling Technology, 13820S), guinea pig anti-EOMES (1:500, Asis Biofarm, OB-PGP022); rat anti-EOMES (1:500, Thermo Fisher, 12-4875-82), rabbit anti-ASCL1 (1:1,000, Cosmo Bio, SKT01-003), rabbit anti-ID3 (1:5,000, Biocheck Inc, BCH-4/17-3), rabbit anti-HOPX (1:500, Proteintech, 11419-1-AP), rabbit anti-ID1 (1:2,000, Biocheck Inc, BCH-1/37-2), and chicken anti-GFP (1:3,000, Aves labs, GFP-1020).

### Mouse *Bmp7* mRNA *in situ* hybridization

Mouse *Bmp7* mRNA *in situ* RNA hybridization experiment was performed using digoxigenin riboprobes on 20 μm cryostat sections as described previously (Zhang et al., 2020b). Riboprobes were made from cDNAs amplified by polymerase chain reaction using the following primers: *Bmp7*-F: GGGCCAGAACTGAGTAAAGGAC; *Bmp7*-R: GAAGCTCATGACCATGTCGG.

### Human *BMP7* mRNAscope *in situ* hybridization

Human *BMP7* mRNA *in situ* hybridization on GW18 human brain sections was performed on 60 μm cryostat sections using the RNAscope™ assay as described previously (Wang et al., 2012). The human *BMP7* probe (424401) was purchased from Advanced Cell Diagnostics, Newark, CA. The RNAscope Multiplex Fluorescent Reagent Kit v2 (Advanced Cell Diagnostics, Newark, CA, 323110) was used according to the instruction manual. The slides were dehydrated in a series of ethanol and loaded onto the Leica Bond RX automated stainer, followed by the pretreatments (protease), probe incubation, amplification steps, fluorophores, and DAPI counterstain. Quality control for RNA integrity was completed using probes specific to the housekeeping genes PPIB. Negative control background staining was evaluated using a probe specific to the bacterial DapB gene. The original picture of *BMP7* mRNA *in situ* hybridization on human GW8 cortical section is from Abu-Khalil et al. (2004).

### Western blot

*Bmp7* was delivered (via *pCAG-Bmp7-Gfp)* into the mouse dorsal cortical VZ at E14.5 or E15.5 using IUE. Controls were electroporated with *pCAG-GFP*. The IUE-mouse cortex was collected 48 h later under a microscope. The cortex was lysed with RIPA lysis buffer (Beyotime, P0013K); main components of which are 50 mmol/L Tris (pH 7.4), 150 mmol/L NaCl, 1% Triton-X-100, 1% sodium deoxycholate, 0.1% SDS, and 1% PMSF (Beyond, ST506-2). Protein concentration was determined using a bicinchoninic assay kit (EpiZyme, ZJ101). Equal amounts of protein were loaded onto 8% SDS-PAGE gels, and Western blot was performed with GLI3 antibody (Goat anti-GLI3, 1:1,000, R&D System, AF3690) overnight at 4°C. Then membranes were washed three times with TBST, and secondary antibodies (Horseradish Hydrogen Peroxide Labeled Donkey Anti-Goat IgG (H+L), Beyotime, A0181, 1:1,000) was incubated at RT for 1 h. Next, membranes were washed by TBST and placed in BeyoECL Sta detection solution. Blots were imaged using e-blot biomolecular imager and bands were quantified using ImageJ software. β-Actin rabbit mAb (monoclonal antibody) was from ABClonal, Ac026, and Horseradish Hydrogen Peroxide Labeled Goat Anti-Rabbit IgG (H+L) was from Beyotime, A0208.

### Cortical single-cell dissociation

CellTrace Yellow (Life Technologies, #C34567, 0.5 μL of 10 mmol/L) (Telley et al., 2016; Govindan et al., 2018) was injected into the lateral ventricle of *Smo*^*F*/*F*^ or *hGFAP-Cre*;*Smo*^*F*/*F*^ mice at E17.0. The cortex was collected 24 h later (E18.0) for cell sorting and scRNA-Seq analysis. Briefly, mouse embryos were dissected out and immediately submerged in fresh ice-cold Hanks balanced salt solution (Gibco 12175-095). The cortex was then cut into pieces and dissociated into a single-cell suspension using a Papain Cell Dissociation Kit (Miltenyi Biotec, catalog no. 130-092-628) according to the manufacturer’s instructions. CellTrace Yellow labeled single-cells were sorted using a BD FACSAriaII (BD Biosciences). One pregnant ferret at E39 was anesthetized and embryos were dissected. The bilateral cortex from one embryo were then cut into pieces and dissociated into a single-cell suspension using a Papain Cell Dissociation Kit.

### Construction of 10× Genomic scRNA-Seq libraries and sequencing

The Chromium droplet-based sequencing platform (10× Genomics) was employed to generate scRNA-Seq libraries, following the manufacturer’s instructions (manual document part number: CG00052 Rev C). The cDNA libraries were purified, quantified using an Agilent 2100 Bioanalyzer, and sequenced on an Illumina Hiseq4000. High-quality sequences (clean reads) of samples were processed using Cell Ranger to obtain quantitative information on gene expression. Cellular quality control thresholds were set at 750–5,000 genes and <10% mitochondrial transcripts per cell. After filtering, the number of cells in our dataset was as follows: E18 *Smo*^*F*/*F*^ (control) cortex, 10,524 cells, 1,954 genes/cell; *hGFAP-Cre*;*Smo*^*F*/*F*^ cortex, 7,596 cells, 2,104 genes/cell; E39 ferret cortex, 23,482 cells, 1,759 genes/cell. scRNA-Seq data from E18 *Smo*^*F*/*F*^ (control) and *hGFAP-Cre*;*Smo*^*F*/*F*^ mice, and E39 ferret cortical scRNA-Seq data have been deposited in the Gene Expression Omnibus (GEO) under the accession number GSE221389. On the other hand, E15.5 mouse cortex scRNA-Seq data is from (Di Bella et al., 2021), E78 rhesus monkey visual cortical scRNA-Seq data is from (Micali et al., 2020), and GW14 human cortex scRNA-Seq data is from (Ma et al., 2021), and GW18, GW22, GW23, and GW26 human cortex scRNA-Seq dataset is from (Trevino et al., 2021). Raw read counts were processed using the global scale normalization method Log Normalize. The normalized datasets were then combined using the Find Integration anchor and Integration Data functions. Statistically significant principal components identified by resampling tests were retained for unified manifold approximation and projection (UMAP) analysis. The enriched genes in different clusters were identified by Wilcoxon rank sum test (ad *p* value < 0.05, |log_2_FC| > 0.25). For cluster annotation, we searched for the most comprehensive and reliable cell type markers through an extensive literature review. All these analyzes were performed in the Seurat package v4.0.6.

### Gene Ontology (GO) enrichment analysis

The GO analysis was performed on statistically significant upregulation genes in cortical oRG cells and tRG cells, using the ClusterProfiler, GO.db, DOSE, org Mm.eg.db, org.hs.eg.db packages in R, and the BH algorithm was used to control the *P*-value adjustment. The *P* value of GO item <0.05 is a significant item. Our analysis was performed separately on upregulated genes in human cortical oRG cells only, or upregulated genes in human cortical tRG cells only at GW22–GW26. The results of these analyses are reported in Table S1.

### Microscopy and imaging

All images of stained sections in this study were collected by Olympus VS120 Automated Slide Scanner with X20. Adobe Photoshop software was used to combine multiple visual fields, if needed. Both Adobe Photoshop and Adobe Illustrator were employed to process or adjust images without destroying the original details.

### Cell counts

In this study, all cell counts were performed on the imaged sections collected by Olympus VS120 Automated Slide Scanner with X20. For the quantification of numbers cells in the control cortex, vector-IUE-cortex, or transgenic mouse cortex, cells were always counted within a certain area of known size in the control or experimental cortex. Normally, at least three sections per cortex and three cortices were analyzed.

### Statistical analysis of cell counts

Individual data points in the figure panels were plotted along with the mean and standard error of the mean (SEM). Unpaired Student’s *t-*test was used to calculate the significance of differences between the two condition analyses. One-way analysis of variance followed by the Tukey–Kramer *post-hoc* test was used for assessing the different significance of the analyses containing more than three conditions. Significance is stated as follows: *P* > 0.05 (ns), *P* < 0.05 (*), *P* < 0.01 (**), *P* < 0.001 (***).

## Supplementary Material

pwad036_suppl_Supplementary_Materials

## Data Availability

The E18 *Smo*^*F*/*F*^ (control) and *hGFAP-Cre*;*Smo*^*F*/*F*^ mouse cortex (mainly including RG cells and progenitors), and E39 ferret cortex scRNA-seq data used in this study have been deposited in the Gene Expression Omnibus (GEO) under the accession number GSE221389. All datasets generated in this study are available upon request.

## Abbreviations

bMIPCs: basal multipotent intermediate progenitor cells
CSF: cerebrospinal fluid
DEPC: diethylpyrocarbonate
dnPKA: dominant negative form of protein kinase A
fRG: full span radial glia
GLI3FL: GLI3 full-length
GLI3R: repressor form of GLI3
GO: gene ontology
GPCR: G protein-coupled receptor
GW: gestational week
IPCs: intermediate progenitor cells
IUE: in utero electroporation
oRG: outer radial glia
OSVZ: outer subventricular zone
OXPHOS: oxidative phosphorylation
PFA: paraformaldehyde
PKA: , protein kinase A
PyNs: pyramidal neurons
RG: radial glia
scRNA-Seq: single-cell RNA sequence
SHHN: non-cholesterol-modified SHH
tRG: truncated radial glia
VZ: ventricular zone
