# Supplementary material for: BMP7 expression in mammalian cortical radial glial cells increases the length of the neurogenic period
Source: Protein Cell. 2023 Jun 10;15(1):21–35. doi: 10.1093/procel/pwad036 (PMC10762677; doi:10.1093/procel/pwad036)
Supplement: pwad036_suppl_Supplementary_Materials [file pwad036_suppl_supplementary_materials.zip › PAC-23144-YZG-Supplementary file (1).pdf]

Supplementary Materials

BMP7 expression in mammalian cortical radial glial cells increases the length of the neurogenic period

Zhenmeiyu Li, Guoping Liu, Lin Yang, Mengge Sun, Zhuangzhi Zhang, Zhejun Xu, Yanjing Gao, Xin Jiang, Zihao Su, Xiaosu Li, Zhengang Yang

Corresponding author: Zhengang Yang, yangz@fudan.edu.cn

Table S1. scRNA-Seq analysis of human cortical cells at GW22, GW23 and GW26; oRG cells are cluster 15 and tRG cells are cluster 12. List of upregulation genes in oRG and tRG cells. Gene ontology (GO) analysis of oRG- and tRG-upregulation genes, respectively.

Table S2. scRNA-Seq analysis of E15.5 mouse cortical cells, E39 ferret cortical cells, E78 rhesus monkey visual cortical cells, and human GW14 cortical cells.

Table S3. scRNA-Seq analysis of the human cortex at GW12, GW14, GW18, GW22, GW23, and GW26

Table S4. scRNA-Seq analysis of E18 *Smo* $^{F/F}$ (control) mouse and *hGFAP-Cre; Smo* $^{F/F}$ (*Smo-cko*) mouse cortical RGC and progenitors; Full list of upregulation and downregulation genes in RG cells (cluster 3): *hGFAP-Cre; Smo* $^{F/F}$ vs *Smo* $^{F/F}$ (control).

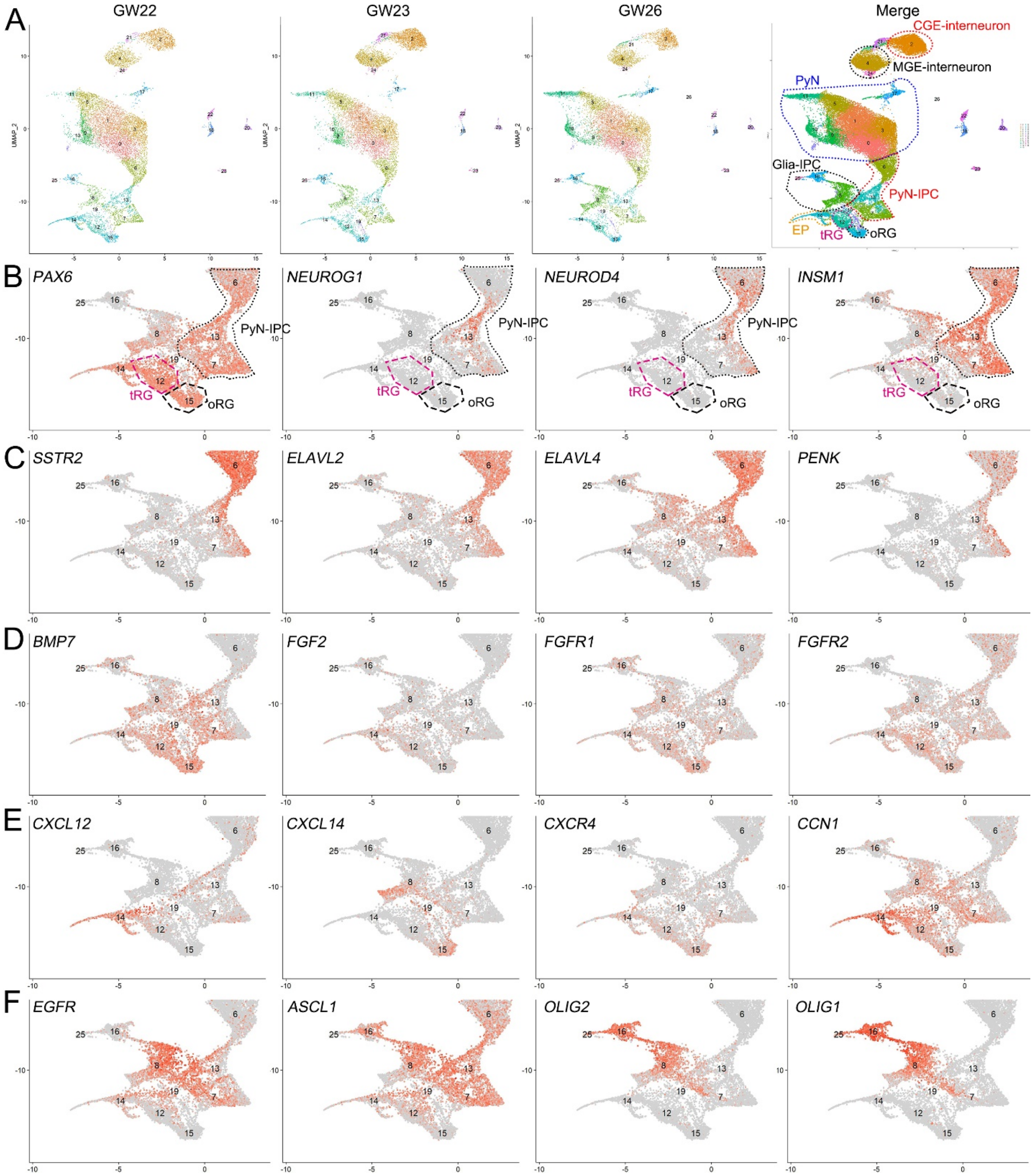

Fig. S1. Human cortical neurogenesis extends to GW26. (A) scRNA-Seq analysis of the developing human cortex at GW22, GW23 and GW26 (also see table S1); the scRNA-Seq data is from Trevino et al., 2021 (PMID: 34390642). (B-F) Feature plots of marker genes; most of these genes are also shown in the heat map (Fig. 1E).

Ribosome Biogenesis (tRG cells vs. oRG cells)

large ribosomal subunit genes

A	B	C	D
Gene	p_val	p_val_adj	UP
RPL10	2.87219E-88	6.51414E-84	UP
RPL10A	1.322E-76	2.99831E-72	UP
RPL11	3.94E-84	8.93E-80	UP
RPL12	8.44E-55	1.92E-50	UP
RPL13	3.20E-92	7.26E-88	UP
RPL13A	1.35E-75	3.06E-71	UP
RPL14	1.75E-52	3.97E-48	UP
RPL15	3.09E-49	7.01E-45	UP
RPL18	6.11E-102	1.39E-97	UP
RPL18A	9.79E-79	2.22E-74	UP
RPL19	3.87E-92	8.78E-88	UP
RPL21	6.24E-82	1.41E-77	UP
RPL22	7.82E-71	1.77E-66	UP
RPL23	4.91E-59	1.11E-54	UP
RPL23A	1.77E-62	4.00E-58	UP
RPL24	6.87E-73	1.56E-68	UP
RPL26	2.74E-91	6.21E-87	UP
RPL27	3.47E-48	7.87E-44	UP
RPL27A	2.87E-68	6.51E-64	UP
RPL28	3.82E-56	8.66E-52	UP
RPL29	2.72E-91	6.16E-87	UP
RPL3	5.05E-115	1.14E-110	UP
RPL30	1.03E-79	2.34E-75	UP
RPL31	1.21E-40	2.75E-36	UP
RPL32	5.37E-109	1.22E-104	UP
RPL34	5.37E-111	1.22E-106	UP
RPL35	1.04E-28	2.36E-24	UP
RPL35A	9.62E-90	2.18E-85	UP
RPL36	2.53E-36	5.73E-32	UP
RPL36A	6.47E-15	1.47E-10	UP
RPL37	9.07E-65	2.06E-60	UP
RPL37A	6.76E-63	1.53E-58	UP
RPL38	1.54E-46	3.49E-42	UP
RPL39	6.31E-89	1.43E-84	UP
RPL4	6.86E-51	1.55E-46	UP
RPL41	2.89E-53	6.56E-49	UP
RPL5	7.78E-77	1.76E-72	UP
RPL6	2.76E-86	6.27E-82	UP
RPL7	1.94E-70	4.39E-66	UP
RPL7A	1.58E-88	3.58E-84	UP
RPL8	6.67E-72	1.51E-67	UP
RPL9	6.16E-76	1.40E-71	UP
RPLP0	3.55E-48	8.06E-44	UP
RPLP1	1.77E-87	4.00E-83	UP
RPLP2	5.62E-54	1.27E-49	UP

small ribosomal subunit genes

A	B	C	D
Gene	p_val	p_val_adj	UP
RPS10	7.16E-12	1.62E-07	UP
RPS11	4.25E-91	9.63E-87	UP
RPS12	6.55E-96	1.49E-91	UP
RPS13	1.12E-90	2.53E-86	UP
RPS14	6.99E-86	1.59E-81	UP
RPS15	1.92E-65	4.35E-61	UP
RPS15A	2.16E-78	4.91E-74	UP
RPS16	1.44E-96	3.27E-92	UP
RPS18	3.45E-91	7.83E-87	UP
RPS19	1.49E-61	3.37E-57	UP
RPS2	4.57E-73	1.04E-68	UP
RPS20	1.85E-16	4.20E-12	UP
RPS21	1.15E-43	2.61E-39	UP
RPS23	1.00E-106	2.28E-102	UP
RPS24	5.56E-91	1.26E-86	UP
RPS25	1.72E-77	3.91E-73	UP
RPS26	1.54E-21	3.50E-17	UP
RPS27	6.74E-64	1.53E-59	UP
RPS27A	5.31E-101	1.20E-96	UP
RPS28	1.97E-31	4.47E-27	UP
RPS29	1.37E-33	3.11E-29	UP
RPS3	3.17E-104	7.18E-100	UP
RPS3A	4.11E-111	9.32E-107	UP
RPS4X	8.97E-87	2.03E-82	UP
RPS5	1.51E-59	3.42E-55	UP
RPS6	7.26E-90	1.65E-85	UP
RPS7	8.19E-55	1.86E-50	UP
RPS8	9.80E-79	2.22E-74	UP
RPS9	2.15E-94	4.87E-90	UP
RPSA	1.88528E-42	4.27581E-38	UP

OXPHOS structure genes

Complex I mtDNA genes

ND1	5.22432E-38	1.18488E-33	UP
ND2	1.05189E-34	2.38569E-30	UP
ND3	4.26095E-32	9.66384E-28	UP
ND4	5.64145E-23	1.27948E-18	UP
ND4L	6.19635E-10	1.40533E-05	UP
ND5	7.46082E-05	1	UP

Complex IV mtDNA genes

COX1	1.46808E-43	3.3296E-39	UP
COX2	9.54852E-70	2.1656E-65	UP
COX3	1.47741E-43	3.35075E-39	UP

Fig. S2. Ribosomal subunit genes are upregulated in tRG cells compared to oRG cells in the human cortex at GW22, GW23, and GW26. Ribosomal subunit genes, mitochondrial genes encoding subunits of the enzyme NADH dehydrogenase, *MT-ND1-5* (*ND1-5*, the respiratory chain Complex I), and mitochondrial genes encoded subunits (*MT-CO1-3, COX1-3*) of respiratory complex IV are significantly upregulated in tRG cells.

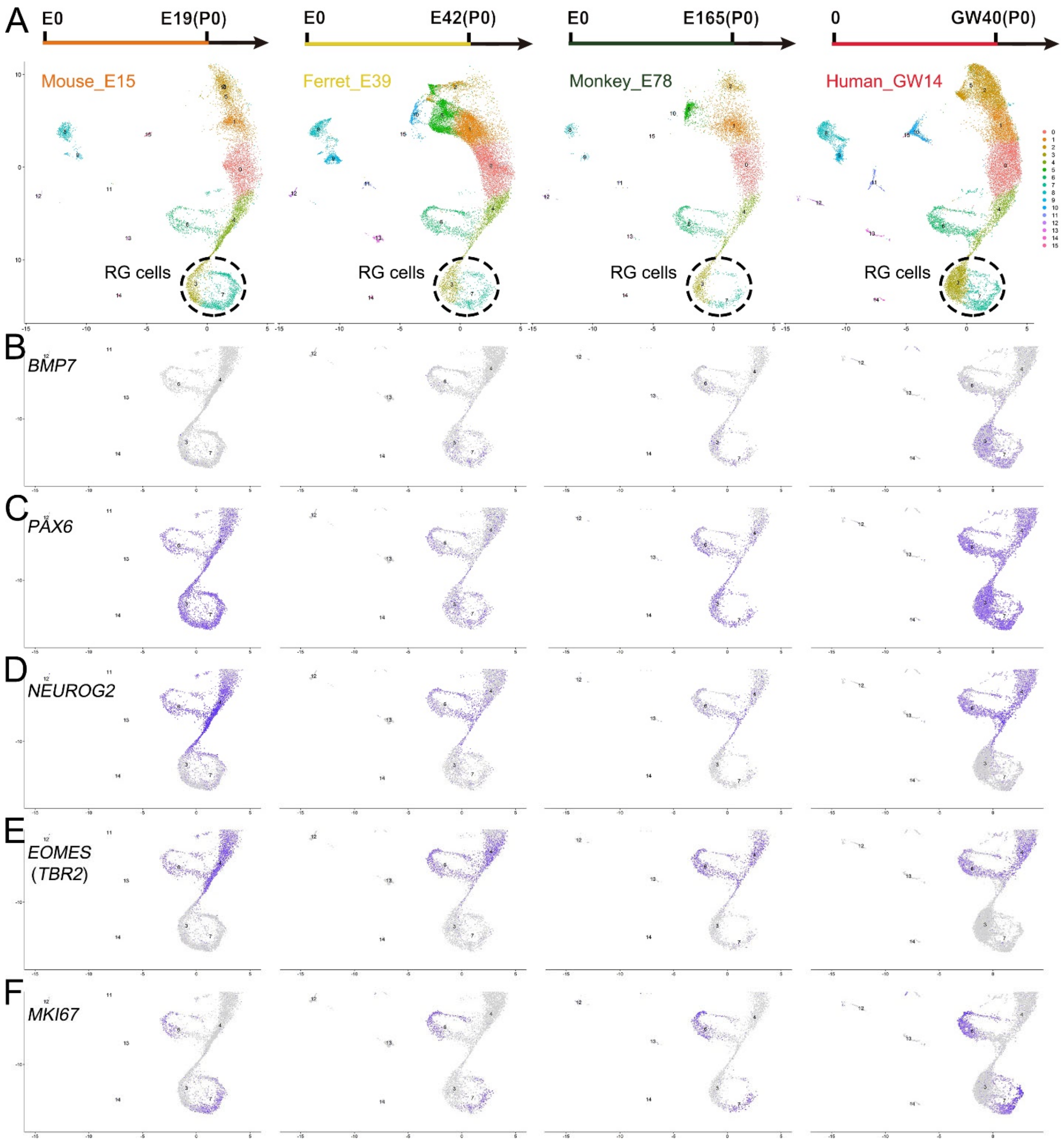

Fig. S3. scRNA-Seq analyses of the developing cortex of mouse, ferret, rhesus monkey and human at the neurogenic stage. (A) Cross-species analyses of transcriptomic signatures of cortical cells. E15.5 mouse cortical scRNA-Seq data is from Di Bella et al., 2021 (PMID: 34163074). E39 ferret cortical scRNA-Seq data was generated in this study, and has been deposited in the Gene Expression Omnibus (GEO) under the accession number GSE221389. E78 rhesus monkey visual cortical scRNA-Seq data is from Micali et al., 2020 (PMID: 32375049), and human GW14 cortical scRNA-Seq data is from Ma et al., 2021 (PMID: 34558085); also see table S2. (B-F) Feature plots of marker genes in different cell types.

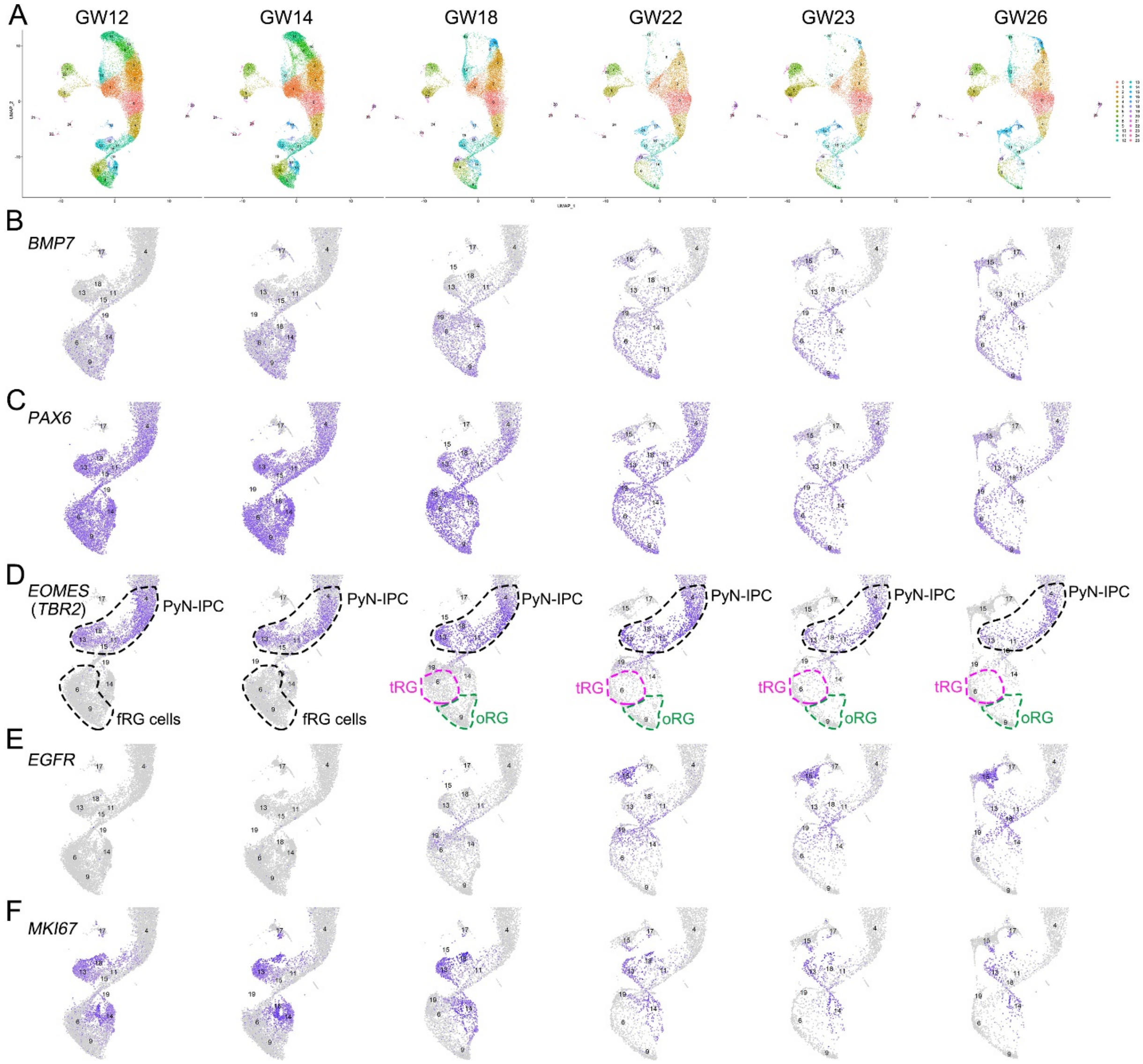

Fig. S4. More and more human cortical RG cells express *BMP7* with increasing gestational age. (A) scRNA-Seq analysis of the developing human cortex. GW12 and GW14 cortical scRNA-Seq datasets are from Ma et al., 2021 (PMID: 34558085), and GW18-GW26 scRNA-Seq datasets are from Trevino et al., 2021 (PMID: 34390642); also see table S3. (B) Feature plots of genes expressed by RG cells (including fRG, oRG, and tRG cells), PyN-IPCs and bMIPCs.

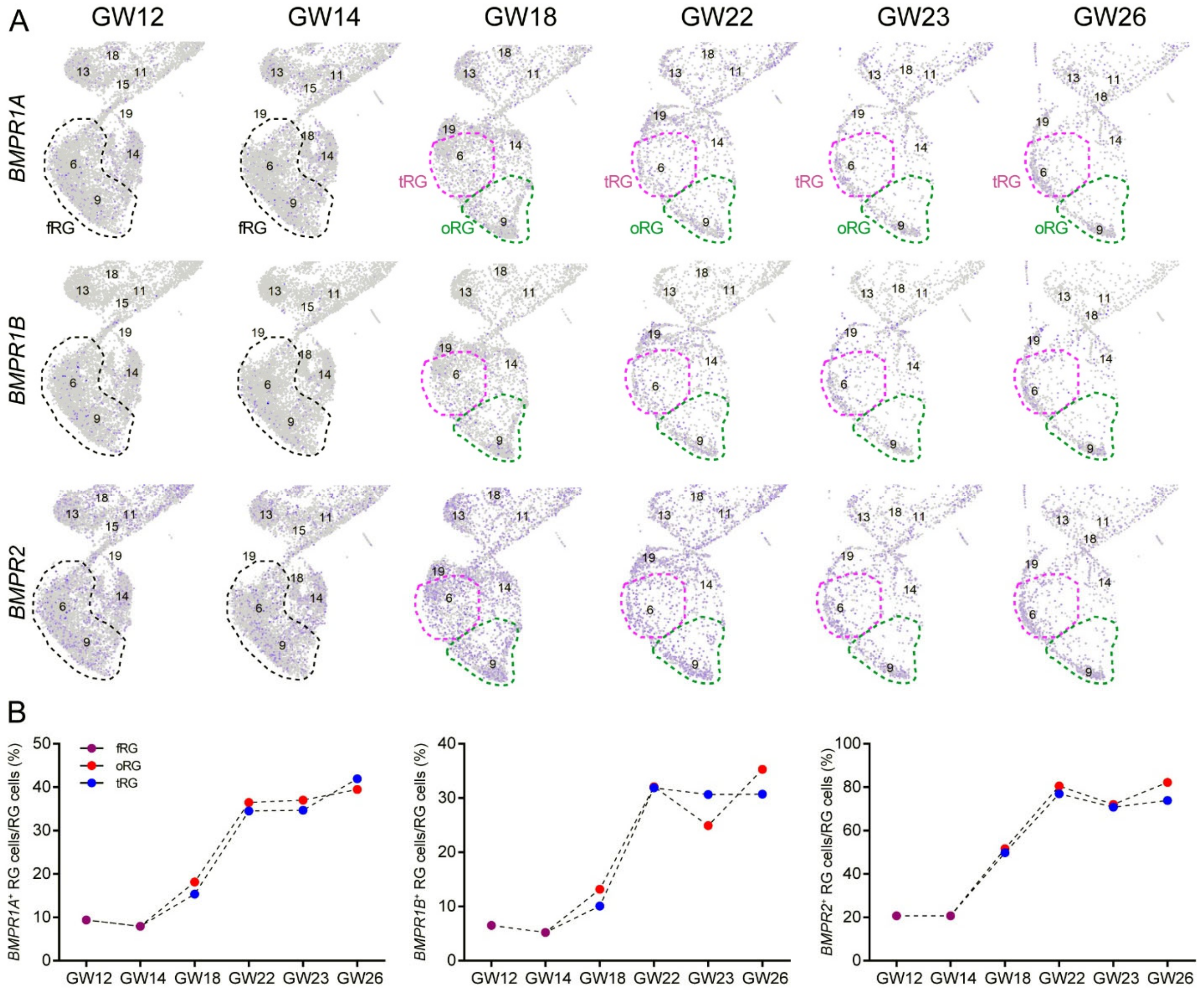

Fig. S5. More and more human cortical RG cells express BMP receptors *BMPR1A, BMPR1B, and BMPR2* with increasing gestational age. (A) Feature plots of *BMPR1A, BMPR1B, and BMPR2* expression in human cortical RG cells (including fRG, oRG, and tRG cells), PyN-IPCs and bMIPCs. (B) Increasing the percentage of human cortical RG cells that expressed *BMPRs* with increasing gestational age.

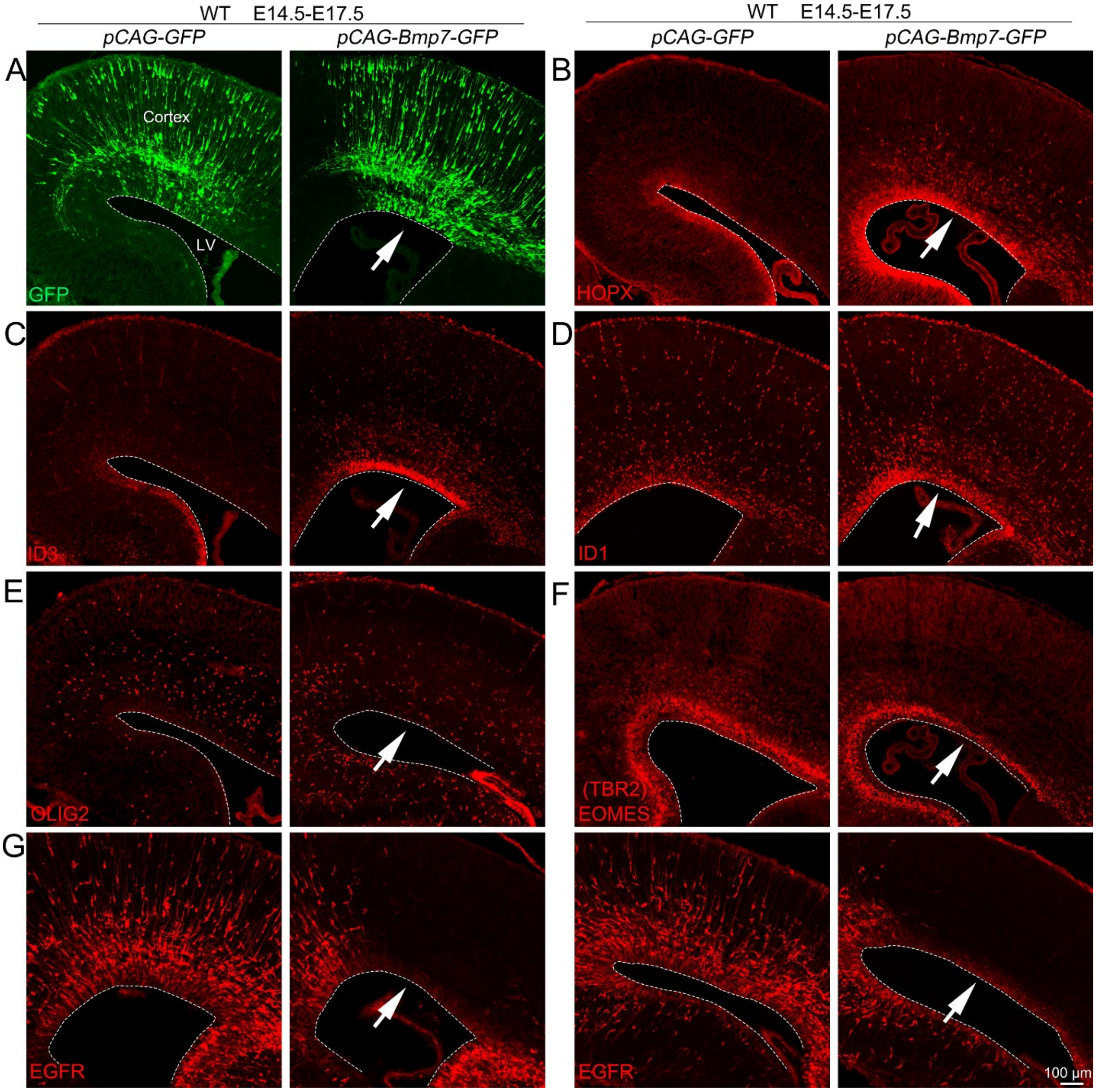

Fig. S6. UP and down regulation of BMP7 response genes following *BMP7* overexpression in the cortex. (A-G) Overexpression of *pCAG-Bmp7-GFP* in the wild type (WT) CD-1 mouse cortex using IUE significantly increases the expression of HOPX, ID3 and ID1, but decreases the expression of OLIG2, EOMES (TBR2), and EGFR (arrows) compared to the *pCAG-GFP*-IUE (control) cortex.

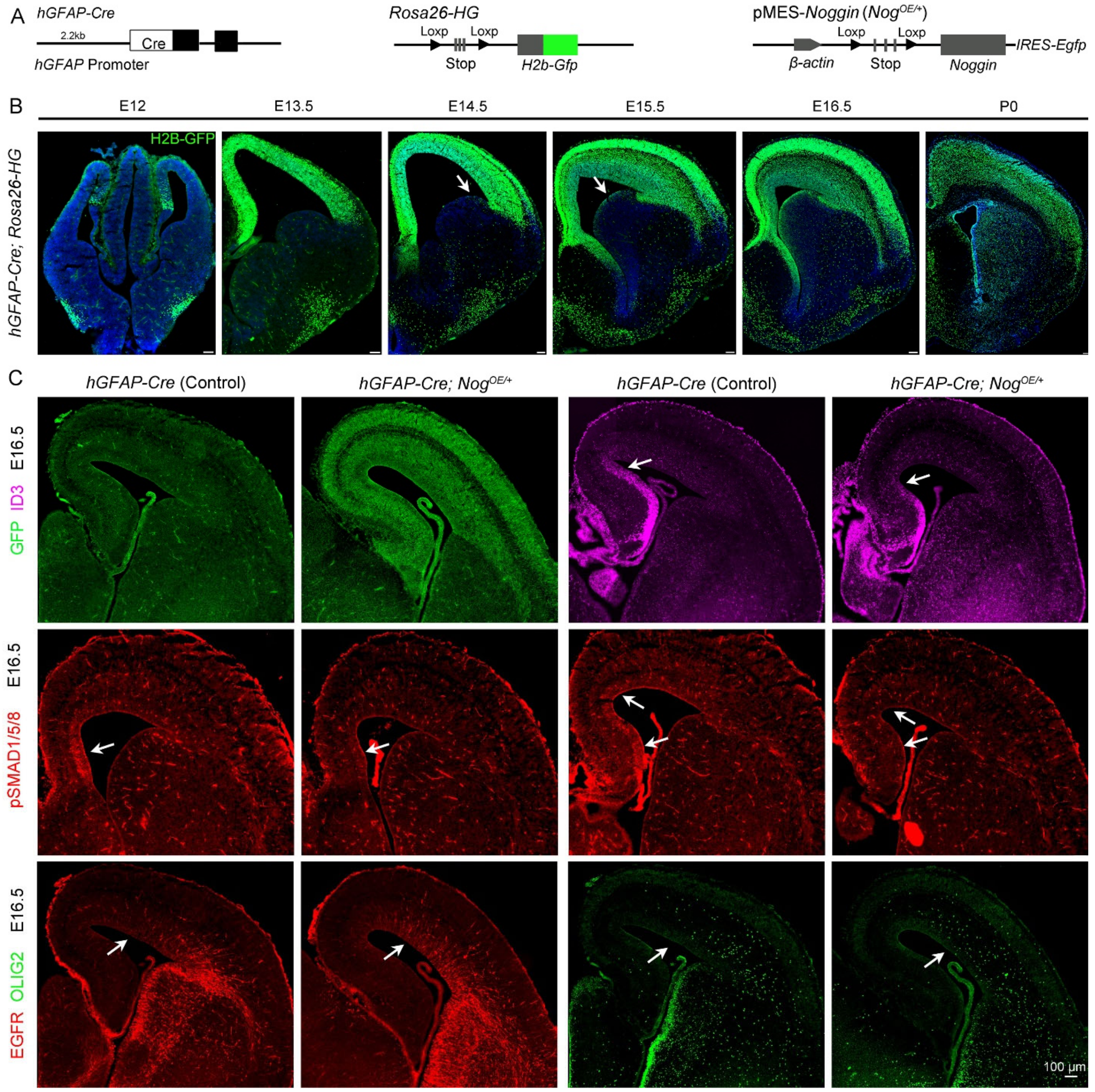

Fig. S7. Inhibition of the BMP function promotes cortical gliogenesis. (A)Three mouse lines are listed. (B) *hGFAP-Cre* is expressed in the mouse medial cortical RG cells at E12. Subsequently, *hGFAP-Cre* is expressed by virtually all cortical RG cells. Note that *hGFAP-Cre* expression begins in RG cells in the LGE (lateral ganglionic eminence) from ~E15.5 (arrows). (C) The expression of ID3 and pSMAD1/5/9 was decreased, whereas the expression of EGFR and OLIG2 was increased in the medial and/or dorsal cortex of *hGFAP-Cre; Nog*$^{OE/+}$ double transgenic mice (arrows).

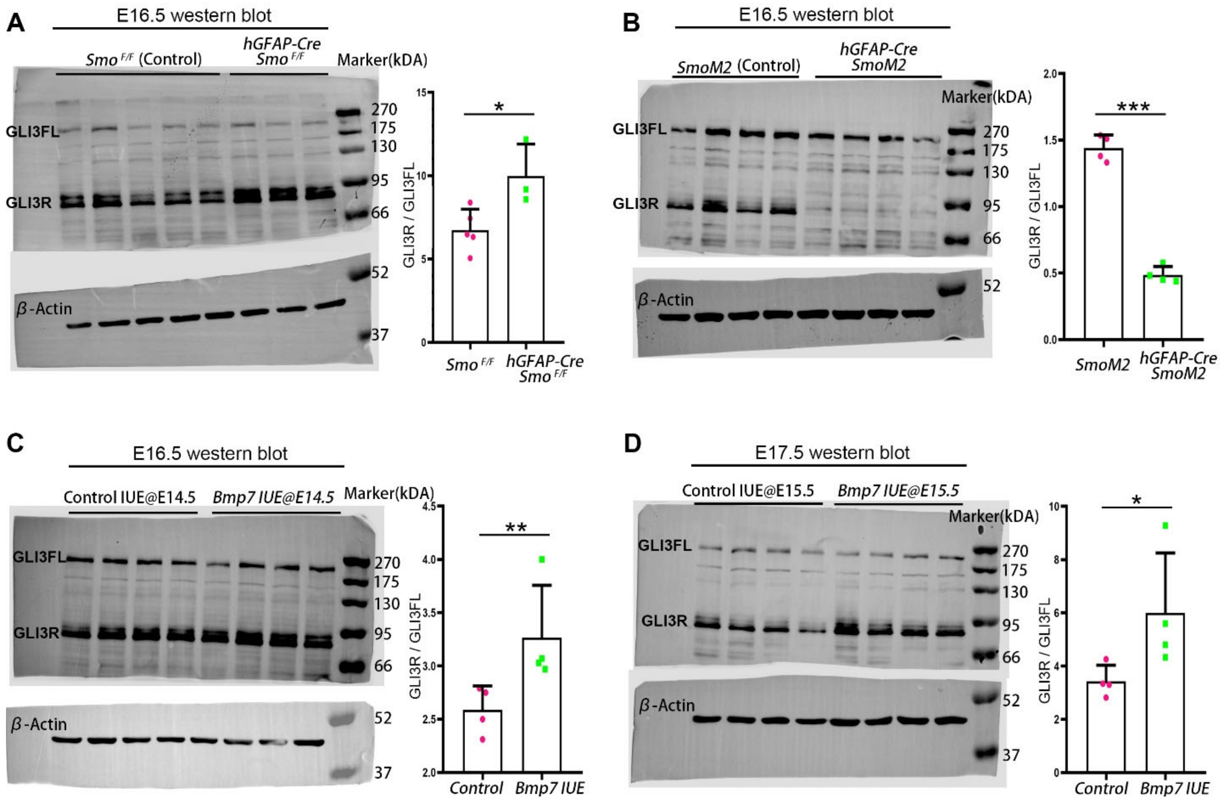

Fig. S8. Western blot analysis reveals that SHH-Smo signaling decreases while BMP7 increases the production of GLI3R in the mouse cortex. (A) Experiment 1: the ratio of GLI3R/GLI3FL was significantly increased in the cortex of *hGFAP-Cre; Smo*$^{F/F}$ mice compared to control mice. (B) Experiment 2: the ratio of GLI3R/GLI3FL was significantly decreased in the cortex of *hGFAP-Cre; SmoM2* mice. (C, D) Experiment 3 and 4: overexpression of *Bmp7* in the cortex using IUE at E14.5 or E15.5, and the IUE-cortex was collected 48 hours later. Western blot showed that the ratio of GLI3R/GLI3FL was significantly increased.

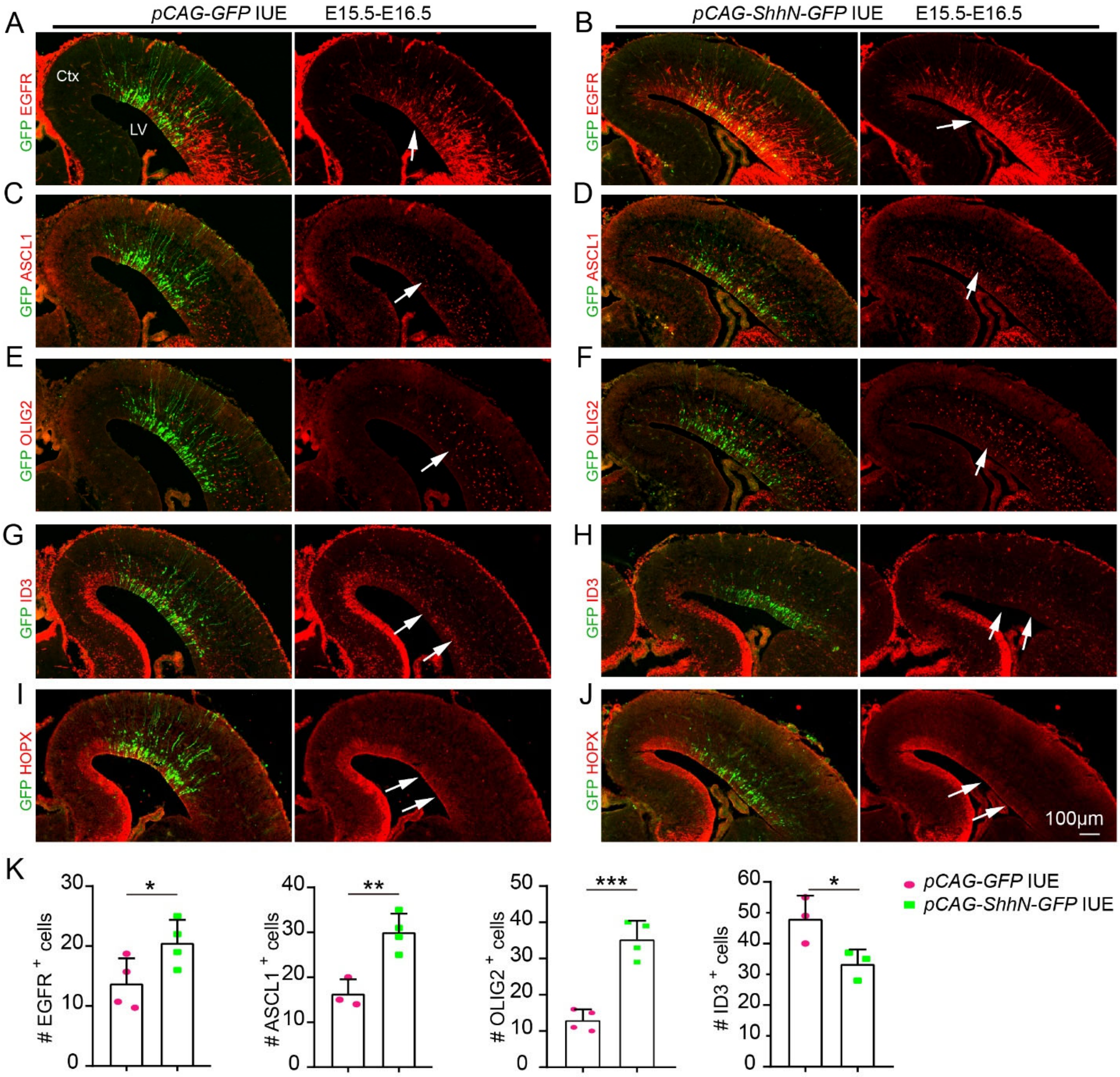

Fig. S9. *ShhN* overexpression promotes cortical gliogenesis. (A-K) Overexpression of non-cholesterol-modified SHH (SHHN) in the cortical VZ by IUE of *pCAG-ShhN-GFP* at E15.5 and the cortex was examined at E16.5. The expression of EGFR, ASCL1 and OLIG2 was increased in the *ShhN*-IUE cortex (arrows). In contrast, expression of ID3 and HOPX was decreased in the *ShhN*-IUE cortex compared to the *GFP*-IUE (control) cortex (arrows). Ctx, cortex.

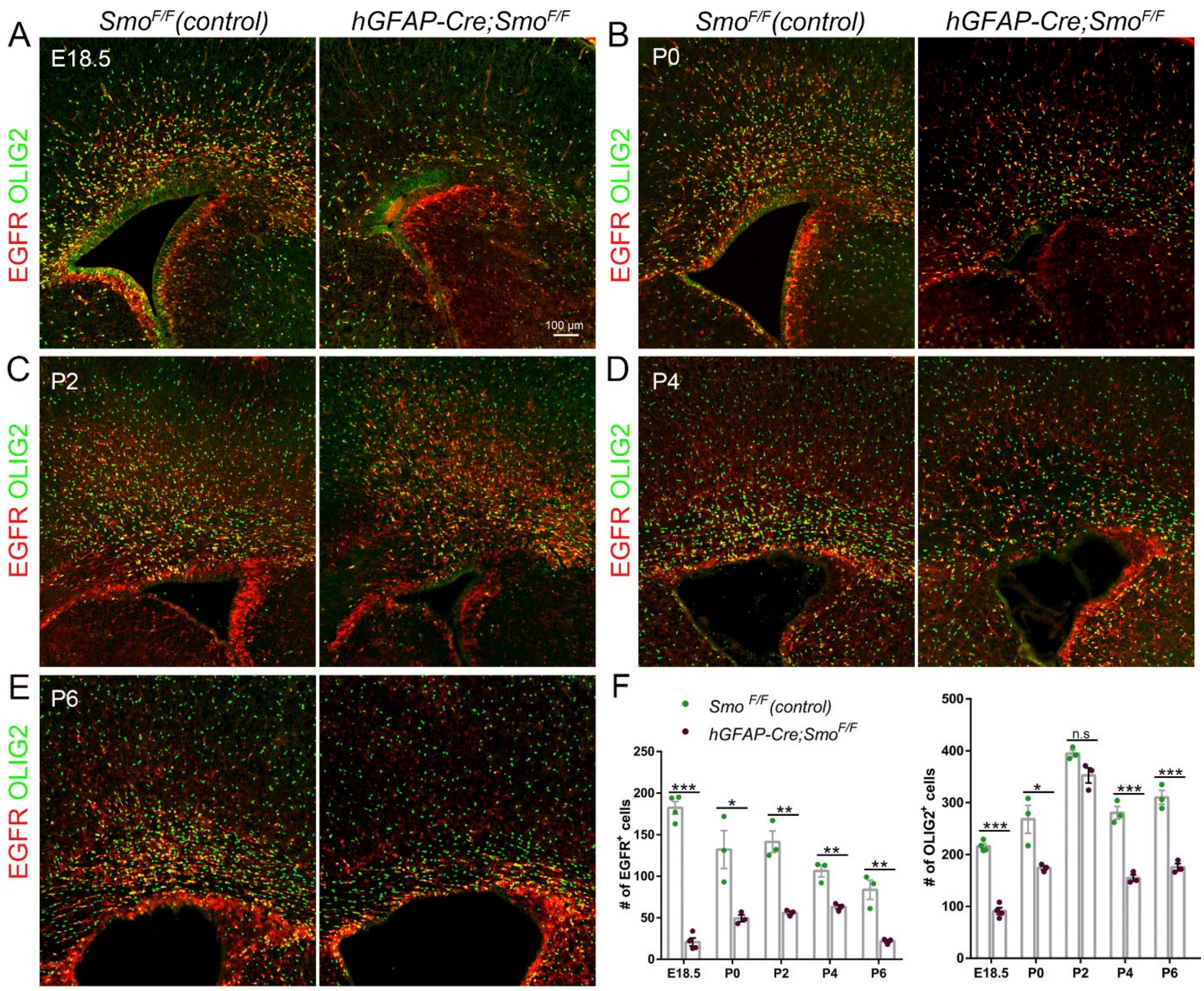

Fig. S10. Cortical gliogenesis is retarded without SHH-SMO function. (A-F) The expression of EGFR and OLIG2 is significantly reduced in the cortex of *hGFAP-Cre; Smo*$^{F/F}$ mice. Note that EGFR expression was partially recovered in the P2 cortex (including VZ and SVZ) indicating a partial recovery of the gliogenesis in the postnatal cortex of *hGFAP-Cre; Smo*$^{F/F}$ mice.

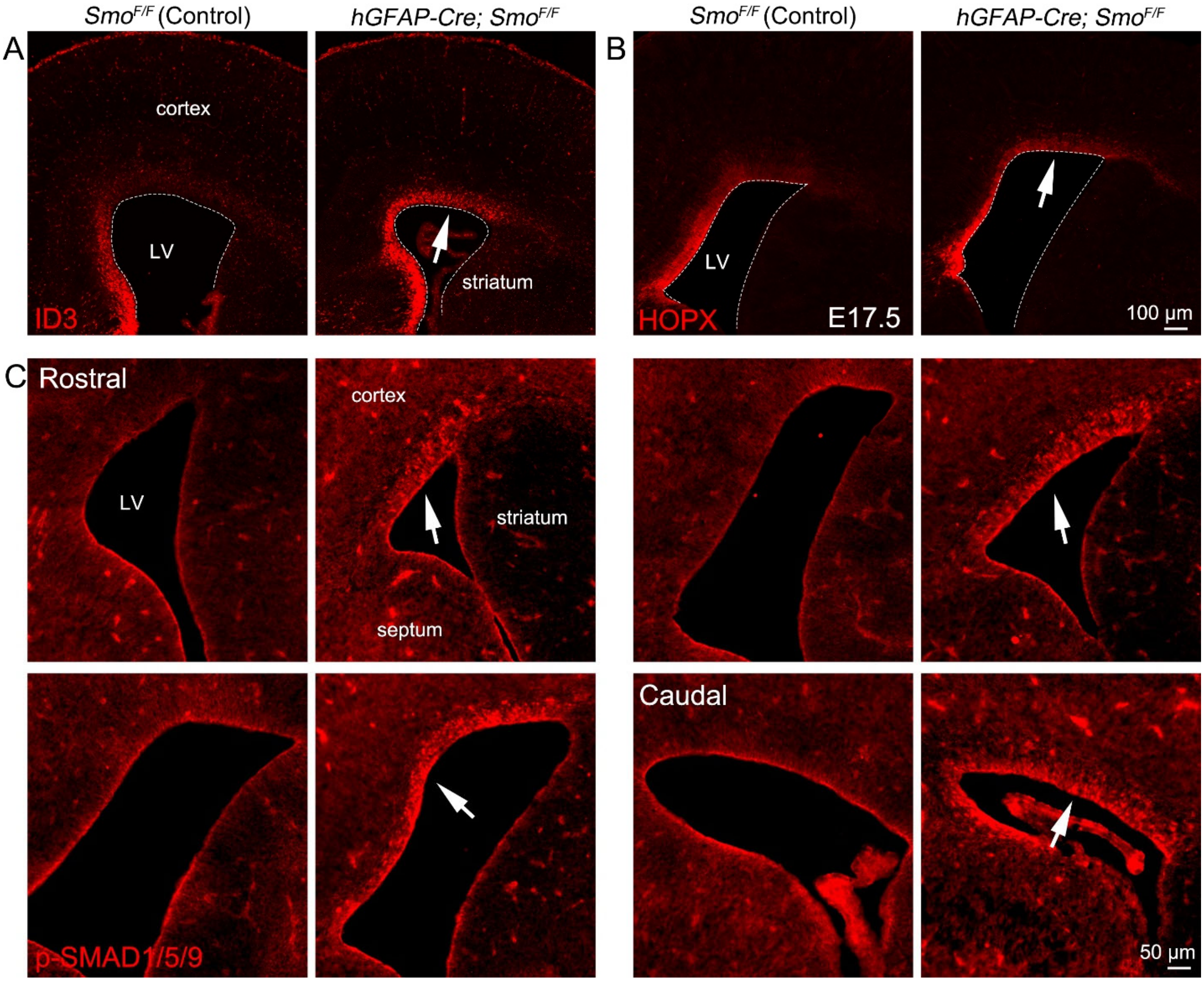

Fig. S11. SHH-Smo signaling inhibits BMP signaling in the cortex. (A-D) The expression of BMP signaling downstream targets ID3, HOPX and pSMAD1/5/9 was significantly upregulated in cortical RG cells of *hGFAP-Cre; Smo*$^{F/F}$ mice at E17.5 (arrows).

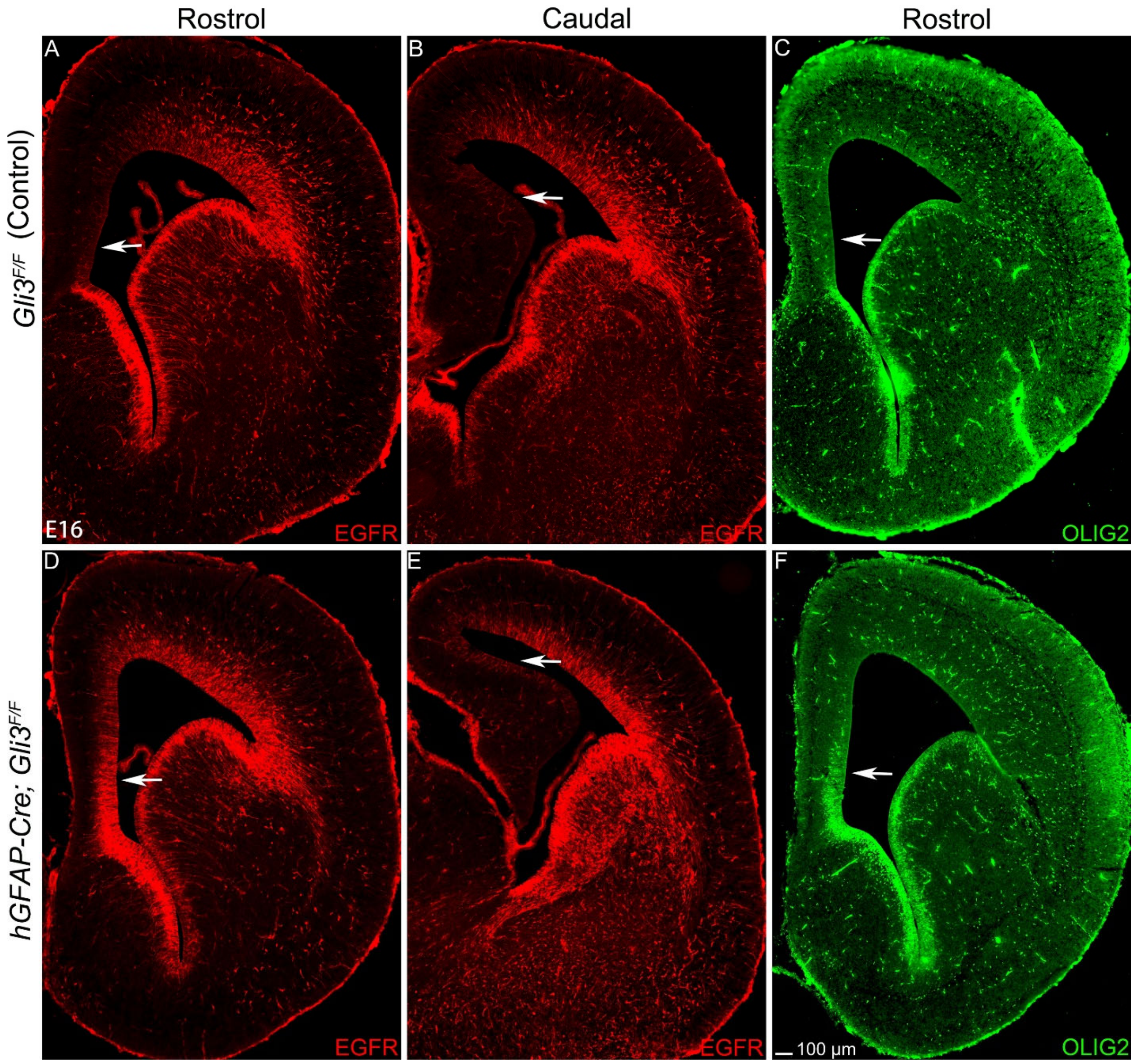

Fig. S12. GLI3R represses EGFR and OLIG2 expression in the cortex. (A-F) The expression of EGFR and OLIG2 was increased in the cortex of *hGFAP-Cre; Gli3*$^{F/F}$ mice at E16 (arrows).

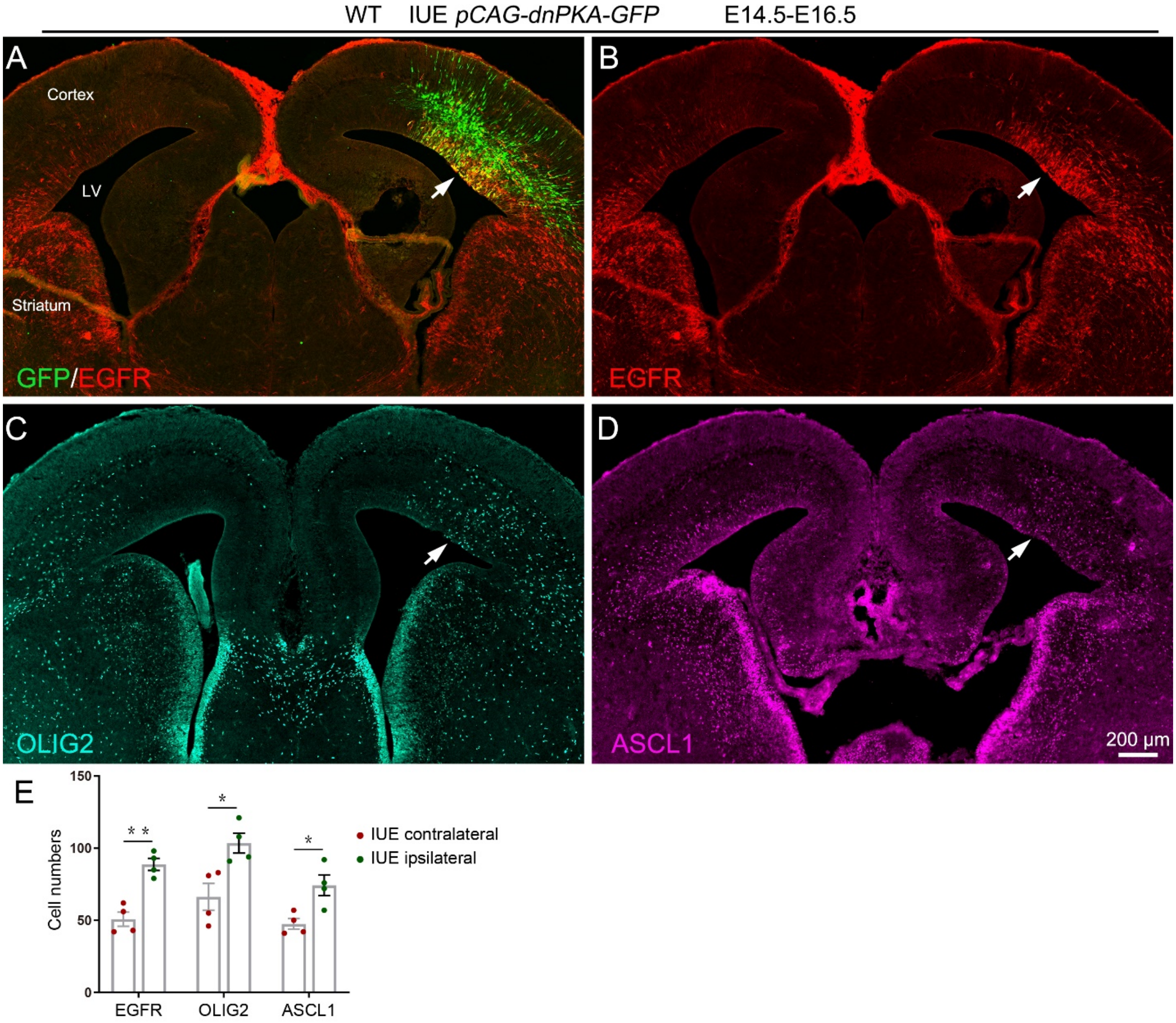

Fig. S13. Overexpression of *dnPKA* using IUE to block GLI3R production results in upregulation of EGFR, OLIG2 and ASCL1 in the wild type mouse cortex. (A-E) The expression of EGFR, OLIG2 and ASCL1 was significantly upregulated in the *dnPKA*-IUE cortex (arrows) compared to the contralateral cortex of wild type (WT) CD-1 mice at E16.5.

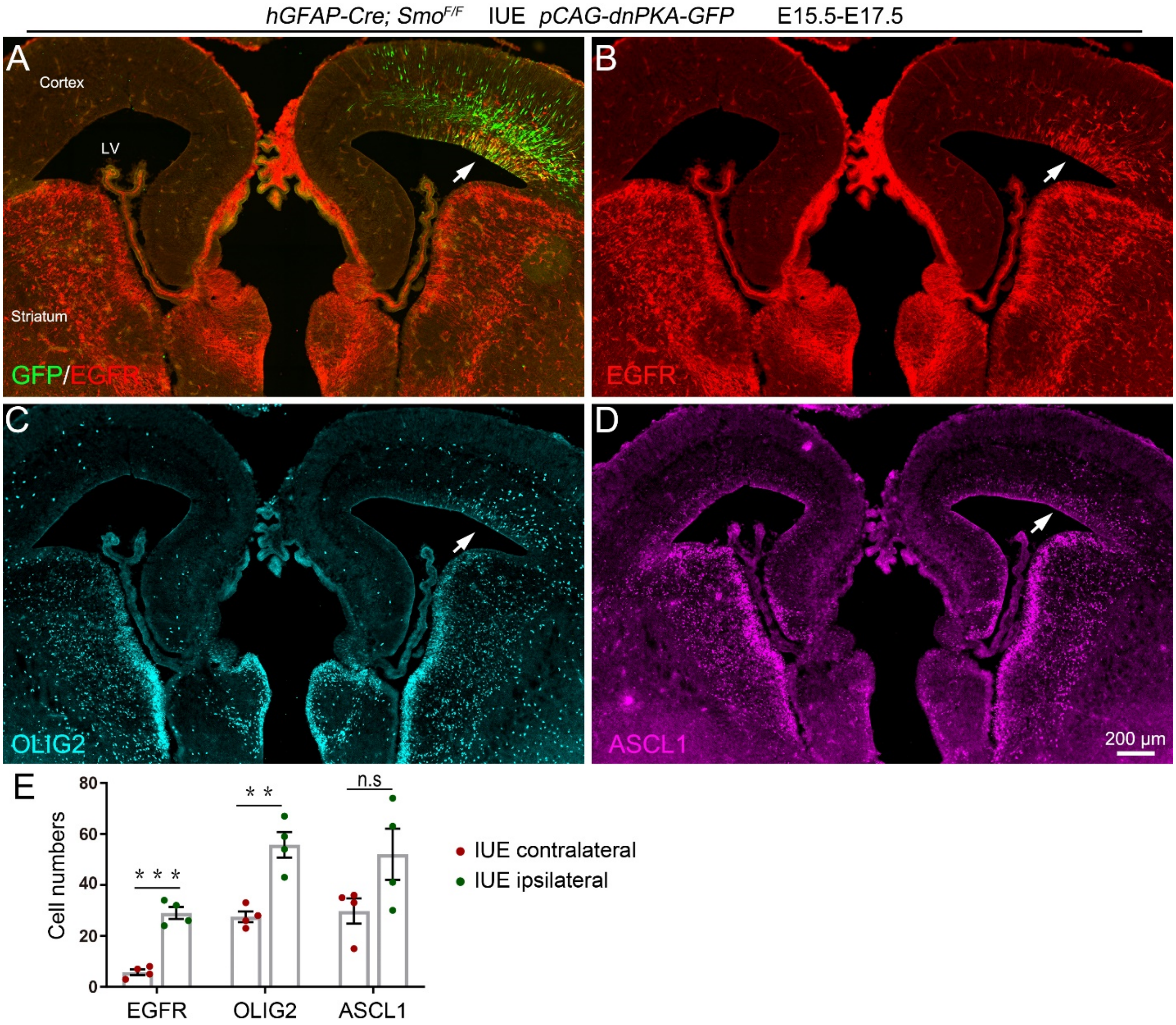

Fig. S14. *dnPKA*-IUE results in upregulation of EGFR, OLIG2 and ASCL1 in the cortex of *hGFAP-Cre; Smo*$^{F/F}$ mice. (A-E) Expression of EGFR, OLIG2 and ASCL1 was significantly increased in the *dnPKA*-IUE cortex (arrows) compared to the contralateral cortex of *hGFAP-Cre; Smo*$^{F/F}$ mice. Note that the expression of EGFR was eliminated in the contralateral cortex of *hGFAP-Cre; Smo*$^{F/F}$ mice at E17.5 (B). A few OLIG2^{+} cells in the contralateral cortex were observed (C), but they were derived from the medial ganglionic eminence.

A

DEGs of RG Cells: hGFAP-Cre; $Smo^{F/F}$ vs. $Smo^{F/F}$

Gene	p-val	avg-log2FC	hGFAP-Cre; $Smo^{F/F}$	$Smo^{F/F}$	p-val_adj	Regulation
Smo	9.32E-18	-0.228	0.002	0.087	1.60E-13	Down
Gli1	2.92E-07	-0.090	0.000	0.030	5.01E-03	Down
Ptch1	1.13E-18	-0.284	0.013	0.115	1.94E-14	Down
Bmp2	1.00E+00	0.000	0.000	0.000	1.00E+00	NS
Bmp5	3.37E-01	-0.003	0.000	0.001	1.00E+00	NS
Bmp6	1.00E+00	0.000	0.000	0.000	1.00E+00	NS
Bmp7	2.58E-05	0.055	0.141	0.074	4.42E-01	Up
Id3	3.81E-53	1.300	0.698	0.351	6.53E-49	Up
Hopx	5.76E-24	0.516	0.911	0.772	9.89E-20	Up
Cdk6	1.04E-21	-0.551	0.176	0.348	1.79E-17	Down
Ccnd1	6.31E-22	-0.535	0.056	0.204	1.08E-17	Down
Cdkn1a	7.61E-50	1.077	0.297	0.040	1.31E-45	Up
Trp53	6.64E-09	0.190	0.483	0.313	1.14E-04	Up
Wnt7b	1.15E-06	0.075	0.107	0.045	1.97E-02	Up
Axin2	1.98E-03	0.036	0.114	0.068	1.00E+00	Up
Lef1	9.72E-11	0.095	0.066	0.009	1.67E-06	Up
Dmrta2	1.75E-11	0.211	0.463	0.274	3.01E-07	Up
Lhx2	3.13E-05	0.119	0.832	0.669	5.38E-01	Up
Emx2	1.75E-14	0.286	0.445	0.244	3.00E-10	Up

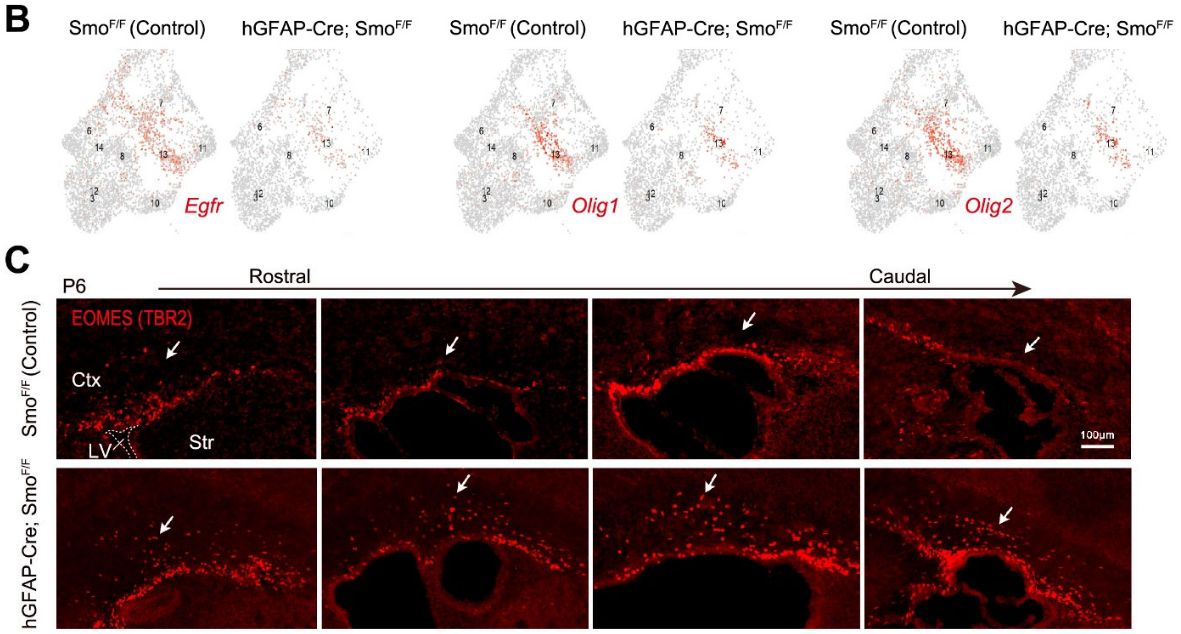

Fig. S15. Loss of *Smo* function in mouse cortical RG cells lengthens the neurogenic period and retards gliogenesis. (A) scRNA-Seq analysis reveals differentially expressed genes (DEG) in E18 cortical RG cells; also see table S4. (B) Numbers of *Egfr*-, *Olig1*- and *Olig2*- expressing cells were significantly reduced in the cortex of *hGFAP-Cre; $Smo^{F/F}$* mice, indicating that cortical gliogenesis is retarded. (C) $EOMES^{+}$ cells were increased in P6 cortex, suggesting that the length of cortical neurogenic period is increased. Note that there were more $EOMES^{+}$ cells in the corpus callosum of *hGFAP-Cre; $Smo^{F/F}$* mice, compared to *$Smo^{F/F}$* (control) mice (arrows). Ctx, cortex; LV, lateral cortex; Str, striatum.

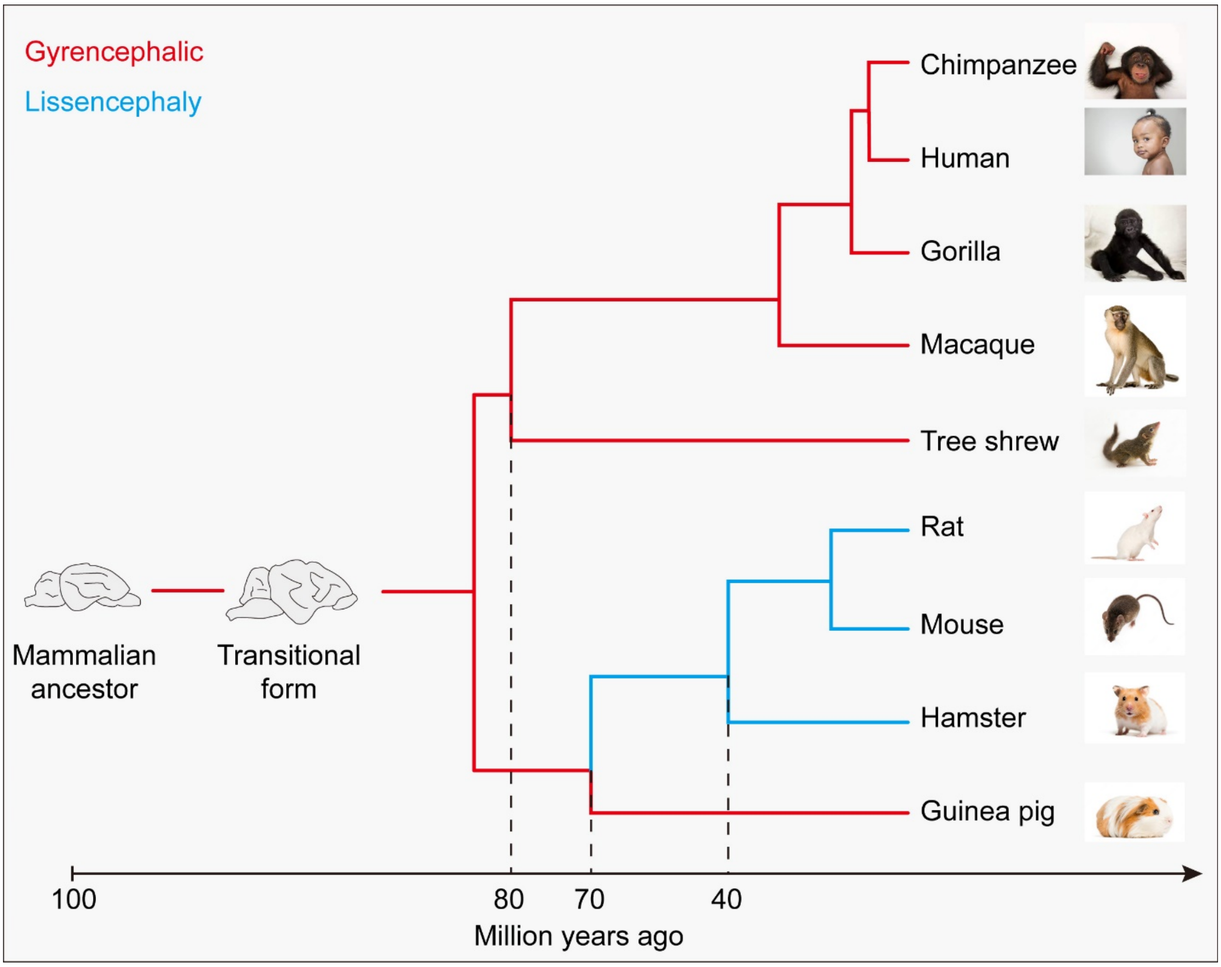

Fig. S16. Evolution of the gyrencephalic and lissencephalic neocortex. Schematic phylogenetic tree illustrating the relationships between the gyrencephalic (red line) and lissencephalic cortex (blue line). Note that the most recent ancestor to all mammals is assumed to have already been gyrencephalic. Therefore, lissencephalic rodents (hamsters, rats and mice) originated from a larger and gyrencephalic ancestor.
